# Therapeutic efficacy of novel memantine nitrate MN‐08 in animal models of Alzheimer’s disease

**DOI:** 10.1111/acel.13371

**Published:** 2021-05-06

**Authors:** Liangmiao Wu, Xinhua Zhou, Yiwan Cao, Shing Hung MAK, Ling Zha, Ning Li, Zhiyang Su, Yifan Han, Yuqiang Wang, Maggie Pui Man Hoi, Yewei Sun, Gaoxiao Zhang, Zaijun Zhang, Xifei Yang

**Affiliations:** ^1^ Department of Neurology and Stroke Center The First Affiliated Hospital of Jinan University and Institute of New Drug Research International Cooperative Laboratory of Traditional Chinese Medicine Modernization and Innovative Drug Development of Chinese Ministry of Education Jinan University College of Pharmacy Guangzhou China; ^2^ Institute of New Drug Research International Cooperative Laboratory of Traditional Chinese Medicine Modernization and Innovative Drug Development of Chinese Ministry of Education Jinan University College of Pharmacy Guangzhou China; ^3^ Department of Applied Biology and Chemical Technology Institute of Modern Chinese Medicine The Hong Kong Polytechnic University Hong Kong China; ^4^ State Key Laboratory of Quality Research in Chinese Medicine and Institute of Chinese Medical Sciences University of Macau Macau China; ^5^ Key Laboratory of Modern Toxicology of Shenzhen Shenzhen Center for Disease Control and Prevention Shenzhen China

**Keywords:** Alzheimer’s disease, cognitive deficits, memantine nitrate, nitric oxide, *N*‐methyl‐D‐aspartate (NMDA) receptors

## Abstract

Alzheimer's disease (AD) is a leading cause of dementia in elderly individuals and therapeutic options for AD are very limited. Over‐activation of N‐methyl‐D‐aspartate (NMDA) receptors, amyloid β (Aβ) aggregation, a decrease in cerebral blood flow (CBF), and downstream pathological events play important roles in the disease progression of AD. In the present study, MN‐08, a novel memantine nitrate, was found to inhibit Aβ accumulation, prevent neuronal and dendritic spine loss, and consequently attenuate cognitive deficits in 2‐month‐old APP/PS1 transgenic mice (for a 6‐month preventative course) and in the 8‐month‐old triple‐transgenic (3×Tg‐AD) mice (for a 4‐month therapeutic course). In vitro, MN‐08 could bind to and antagonize NMDA receptors, inhibit the calcium influx, and reverse the dysregulations of ERK and PI3K/Akt/GSK3β pathway, subsequently preventing glutamate‐induced neuronal loss. In addition, MN‐08 had favorable pharmacokinetics, blood‐brain barrier penetration, and safety profiles in rats and beagle dogs. These findings suggest that the novel memantine nitrate MN‐08 may be a useful therapeutic agent for AD.

## Graphical abstract

Memantine nitrate MN‐08 reverses cognitive impairment by antagonizing NMDA receptor and releasing NO in AD mice. MN‐08 antagonizes NMDA receptor, inhibits calcium influx, attenuates the dysregulations of ERK and PI3K/Akt/GSK3β, reduces Aβ accumulation, and consequently prevents neuronal and dendritic spine loss. Moreover, MN‐08 binds NMDA receptor to release NO, dilates blood vessel and increases cerebral blood flow. These findings suggest that MN‐08 may be a useful therapeutic agent for AD.

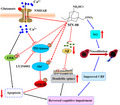

## 1 INTRODUCTION

Alzheimer's disease (AD), one of the most prevailing neurodegenerative disease in the aged population, is characterized by progressive impairment of cognitive function, including the loss of memory, language, executive functions, and social abilities (Walrath & Lawlor, 2019). Aging is the time‐dependent physiological functional decline that is also the most profound risk factor for AD. With the aging population increases globally, the need of effective treatments for AD is rapidly growing. Unfortunately, only limited therapeutic agents are currently available for treating AD.

AD is characterized by overproduction and accumulation of amyloid β (Aβ), Tau hyperphosphorylation, and excitotoxicity, leading to synaptic deficits, all of which might be key factors in neuronal death (Busche et al., 2015; Holtzman et al., 2011; Xie et al., 2013). Glutamate‐induced excitotoxicity has been implicated in excessive activation of *N*‐methyl‐D‐aspartate (NMDA) receptors, which results in overloaded intracellular calcium ions (Ca^2+^), increased free radical production, and formation of Aβ, finally contributing to neuronal death. This is thought to be a critical pathophysiologic mechanism behind the widespread necrosis in the brain and functional impairment seen in dementia patients (Chang et al., 2016; Kemp & McKernan, 2002). Emerging evidence suggests that under most conditions pathological activity is primarily triggered by extrasynaptic NMDA receptors, whereas physiological synaptic NMDA receptors activity regulates neuroprotective molecular pathways in neurons (Hardingham & Bading, 2010; Parsons & Raymond, 2014). Memantine (1‐amino‐3,5‐dimethyladamantane), an open‐channel, non‐competitive NMDA receptor blocker with a fast off‐rate, was approved in 2003 by the U.S. Food and Drug Administration as a symptomatic treatment for moderate to severe AD (Witt et al., 2004), which predominantly blocks extrasynaptic over synaptic NMDA receptors (Xia et al., 2010). However, memantine delivers only partial improvement of AD patient symptoms. Additionally, memantine has been reported to reduce local cerebral blood flow (CBF) in the brain cortices of intact narcotized rats, as well as animals with global transient ischemia induced by occlusion of the middle cerebral artery (Mirzoyan et al., 2014). The CBF of patients with AD may be reduced due to damaged cerebral blood vessels (Iturria‐Medina et al., 2016; Nortley & Korte, 2019). As a result, the supply of oxygen and nutrients to brain tissue decreases, triggering cascading pathophysiologic changes, including the over‐activation of NMDA receptors and neuronal loss.

Nitric oxide (NO), an important secondary messenger in the central nerve system (CNS), is involved in the regulation of CBF in AD (Toda & Okamura, 2012). Several studies show that levels of NO in sera might be decreased in progressive neurodegenerative diseases, especially AD (Corzo et al., 2007). Restoring NO levels by using NO donors in the brain may provide therapeutic benefits for the treatment of cerebral vascular diseases. Currently, NO donors nitroglycerin and sodium nitroprusside are used clinically for treatment of cardiovascular diseases as they systemically dilate blood vessels. These drugs also have been reported to downregulate NMDA receptor activity through an allosteric redox‐modulatory site(s) that is called S‐nitrosylation (Choi et al., 2000; Lipton et al., 1993); however, the short half‐life and poor distribution of traditional NO donors into brain tissue may make them unsuitable for treating AD. Moreover, long‐term usage of these drugs can increase the risk of hypotension in patients by releasing NO into peripheral blood vessels in addition to those in the brain.

**FIGURE 1:**
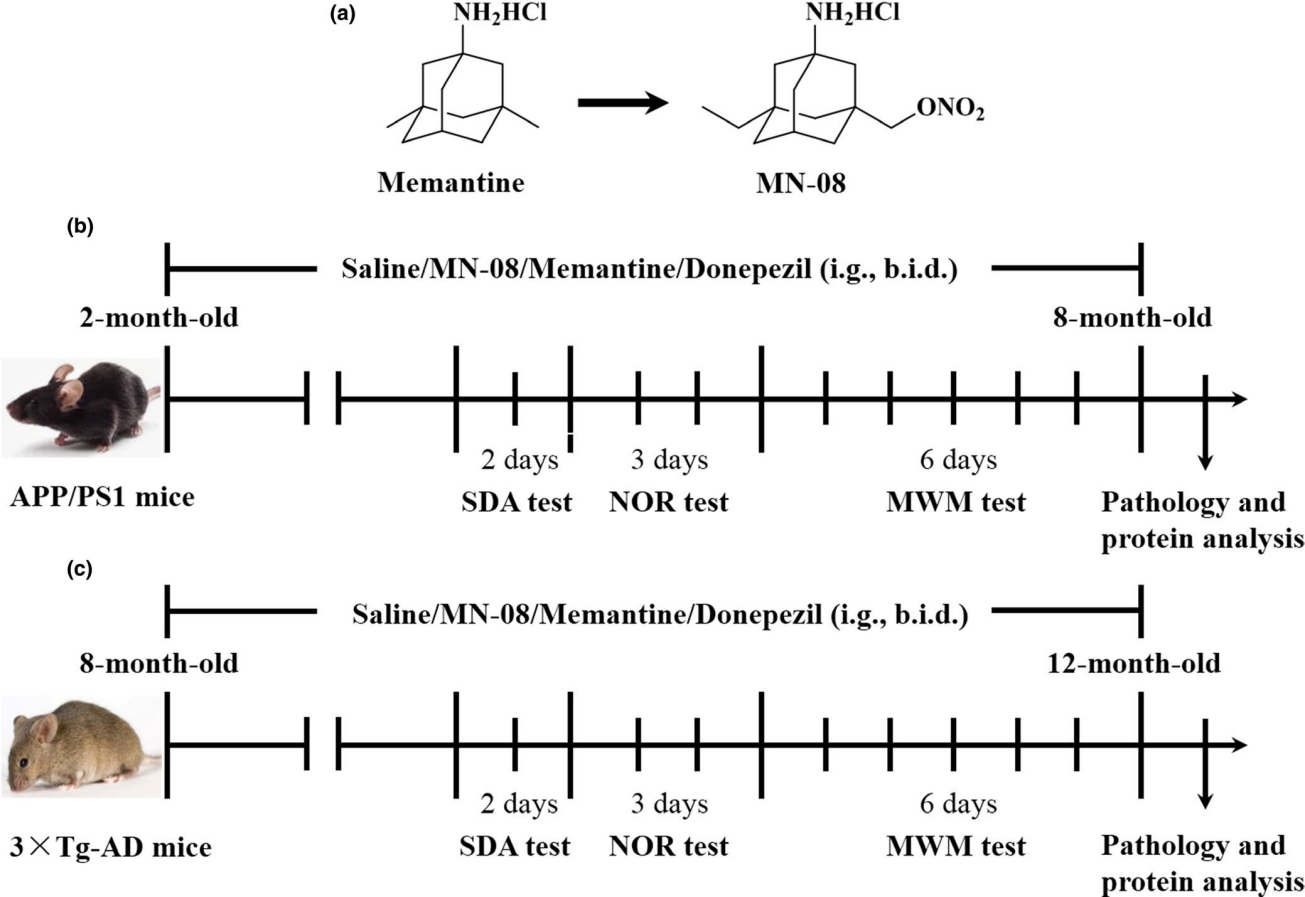
Chemical structures and experimental design for animal models. (a) The chemical structures of memantine and MN‐08. (b) Experimental design of MN‐08 prevention study in APP/PS1 mice. (c) Experimental design of MN‐08 treatment study in 3×Tg‐AD mice. Step‐down avoidance test (SDA test). Novel object recognition test (NOR test). Morris water maze test (MWM test)

NO donors that release NO specifically in brain tissue might avoid inducing side effects secondary to systemic hypotension and be beneficial for treating AD. Additionally, NO‐based compounds preferentially react with allosteric cysteine residues in the NMDA receptor to limit excessive activity (Takahashi et al., 2007). Along these lines, we have previously designed and synthesized memantine nitrates by introducing nitrate group into the major backbone of memantine and reported that it could deliver the NO group to pathologically open NMDAR‐coupled channels by the memantine scaffold binding to the NMDA receptor (Takahashi et al., 2015; Talantova et al., 2013; Tu et al., 2017; Wang et al., 2006). Although memantine nitrates manifested a dual site of action at the NMDA receptors via the open‐channel block and NO/redox modulation of the receptor, all memantine nitrates synthesized by our group were less potent than memantine in the inhibitory effect on NMDA receptors (Takahashi et al., 2015; Wang et al., 2006). Unsuspectedly, memantine nitrates performed in a superior fashion to memantine in treatment of a rat model of stroke (Takahashi et al., 2015; Wang et al., 2006). Recently, we demonstrated that one of the novel memantine nitrates, MN‐08 (structure as shown in Figure 1a), can bind NMDA receptors to release NO, dilate cerebral blood vessels and increase cerebral blood flow, subsequently ameliorating brain injury and cerebral vasospasm in experimental subarachnoid hemorrhage models (Luo et al., 2019); MN‐08 does not, however, change blood pressure in normal rats, lowering the risk of systemic hypotension induced by traditional nitrate drugs (Luo et al., 2019). The effect of MN‐08 on AD is still unclear. In this study, the efficacy and mechanism of action of MN‐08 are examined in two transgenic mouse models of AD, APP/PS1 and triple‐transgenic (3×Tg‐AD) mice, and cells model. Two approved drugs for dementia, namely memantine and donepezil, are compared with MN‐08.

## 2 RESULTS

### 2.1 MN‐08 improves behavioral performance in experimental models of AD

**FIGURE 2:**
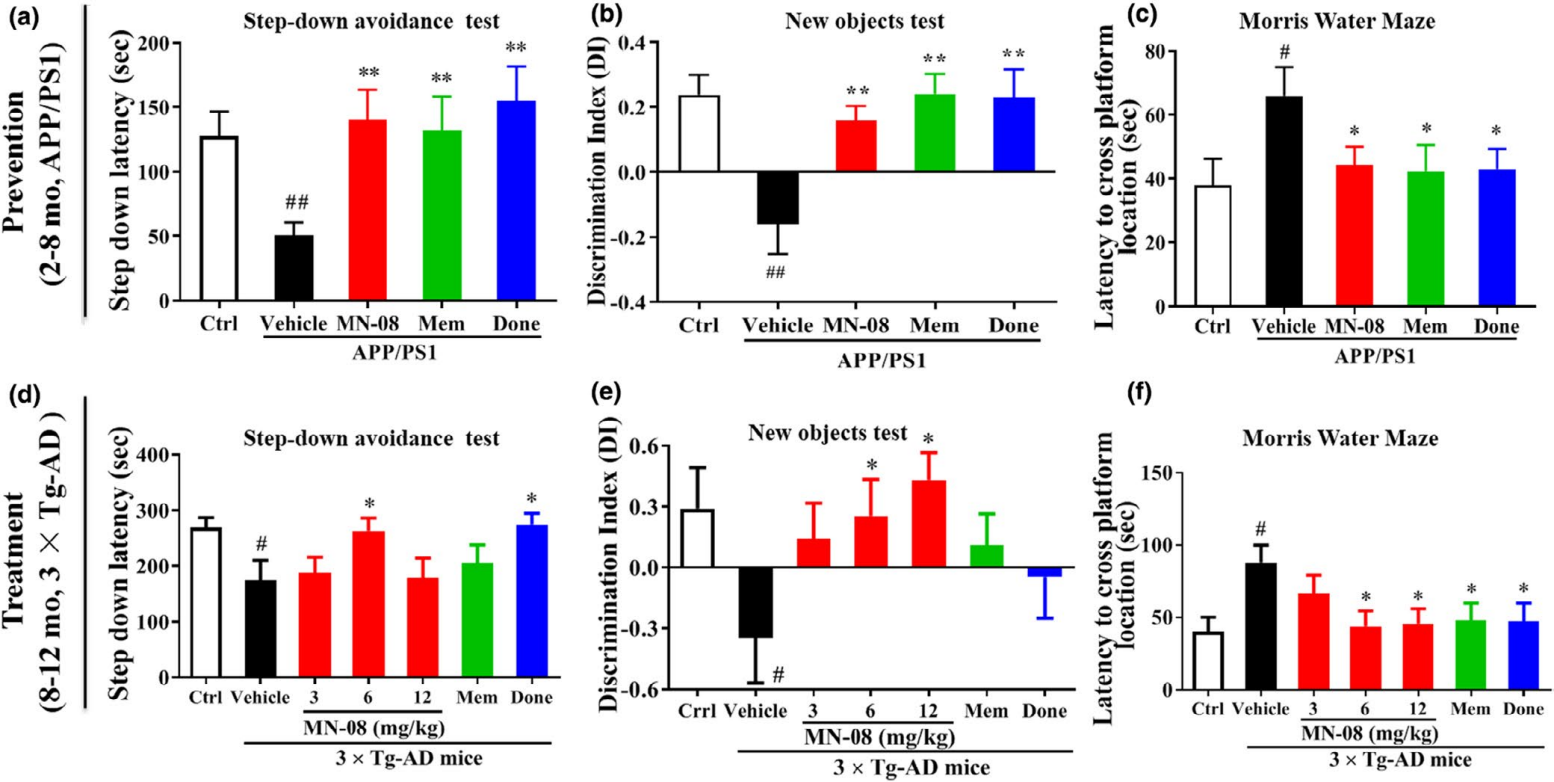
MN‐08 improves behavioral performance in experimental models of Alzheimer's disease. (a–c) Behavioral experiments and statistical analysis of MN‐08’s preventative protocols in 2‐month‐old APP/PS1 mice (*n* = 16–20). (d–f) Behavioral test and quantitative analysis of MN‐08’s therapeutic protocols in 8‐month‐old 3×Tg‐AD mice (*n* = 11–14). (a and d) Latency in the step‐down avoidance (SDA) test. (b and e) The ability to recognize a new object in the novel object recognition (NOR) test. (c and f) Escape latency to find the platform in the Morris water maze (MWM) probe phase (Day 6). Data are presented as mean ± SEM. Significance was determined by one‐way ANOVA followed by Tukey's multiple comparisons test. *^#^p* < 0.05 and *^##^p* < 0.01 vs. control group; *p < 0.05, **p < 0.01 versus vehicle group

We first assessed the preventive effects of MN‐08 on cognitive impairment in APP/PS1 mice. Animals were administered MN‐08 (6 mg/kg) by gastric gavage from ages 2 to 8 months for 6 months successively (Figure 1b). MN‐08 significantly increased the latency in step‐down avoidance (SDA) test and the discrimination index in novel object recognition (NOR) test when compared with vehicle‐treated APP/PS1 mice (Figure 2a,b). In the Morris water maze (MWZ) test, MN‐08 treatment markedly shortened the escape latency and increased the number of crossings in Morris water maze (MWM) test (Figure 2c and Figure S1a–c). These results indicated that the cognitive dysfunction of APP/PS1 mice was ameliorated by a preventive treatment of MN‐08.

Based on the above results, we further evaluated the treatment effects and the dose response of MN‐08 on memory and cognitive disorders in 3×Tg‐AD mice. MN‐08 at doses of 3, 6, and 12 mg/kg were administered to 8‐month‐old 3×Tg‐AD mice for 4 consecutive months (Figure 1c). Compared with vehicle‐treated 3×Tg‐AD mice, animals treated with the different doses of MN‐08 performed better in the SDA, NOR, and MWM tests, as reflected by a significant increase in step‐down latency (Figure 2d), discrimination index (Figure 2e) and the number of crossings (Figure S1f), and a significant reduction in the escape latency time (Figure 2f and Figure S1e). Taken together, these data suggested that MN‐08 can prevent or halt cognitive dysfunction in AD mice.

### 2.2 MN‐08 reduces plaque deposition and the total level of Aβ in APP/PS1 mice

**FIGURE 3:**
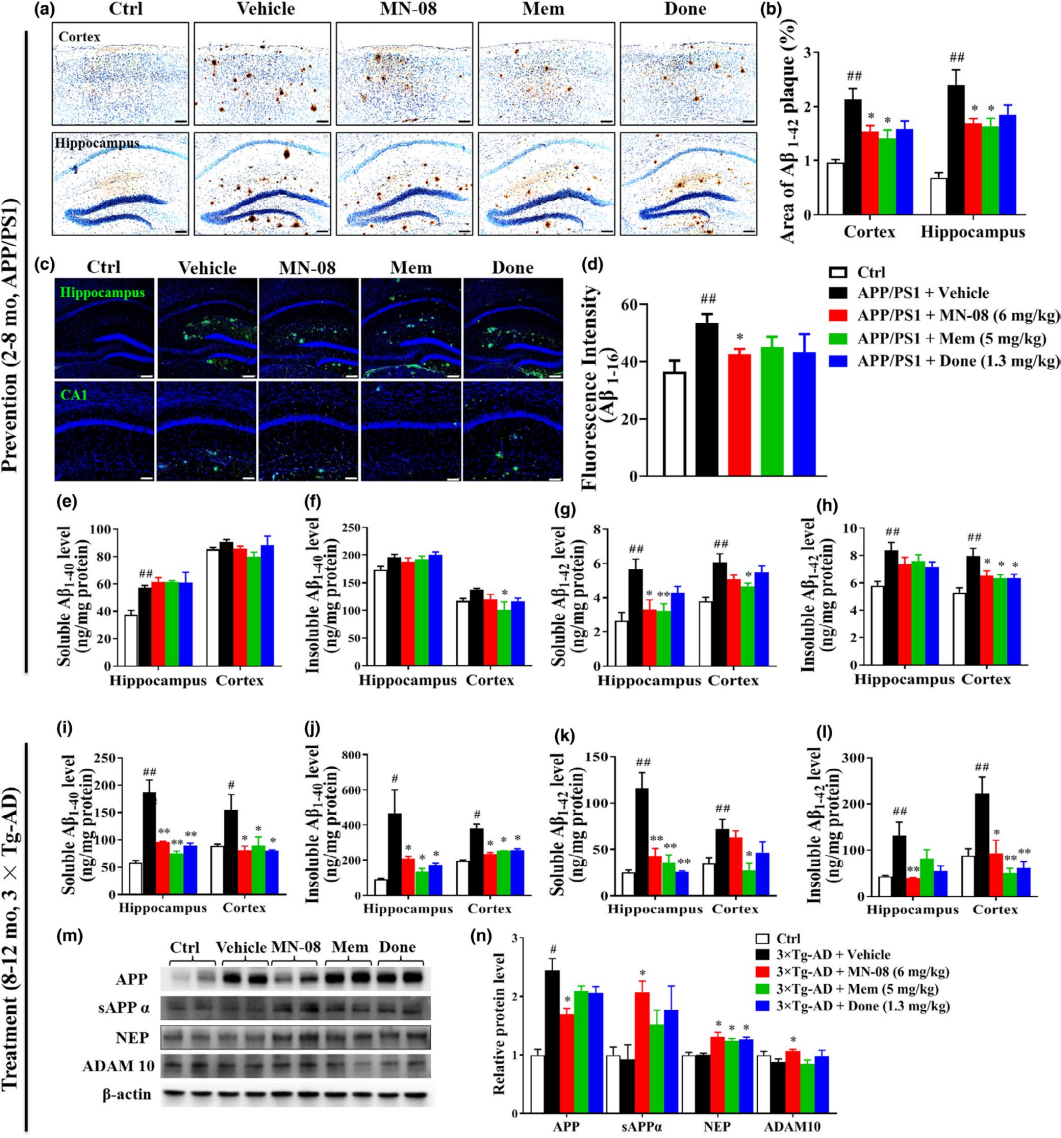
MN‐08 alleviates Aβ burden in AD mice. (a–h) MN‐08 reduces plaque deposition of APP/PS1 mice in the prevention experiment. (a and b) Representative photomicrographs (a) and quantitative analyses (b) of immunohistochemistry staining with Aβ_1‐42_ in the cerebral cortex and hippocampus of control, and animals treated with vehicle, MN‐08 (6 mg/kg), memantine (5 mg/kg), or donepezil. (1.3 mg/kg). Scale bar, 100 μm. (c and d) Representative photomicrographs (c) and quantitative analyses (d) of immunofluorescence staining for Aβ_1‐16_ in hippocampus. Scale bar, 100 μm (upper series) and 50 μm (lower series). (e and f) Total concentration of soluble Aβ_1–40_ (e) and insoluble Aβ_1–40_ (f) in the hippocampi and cortices from APP/PS1 mice. (g and h) Total concentration of soluble Aβ_1–42_ (g) and insoluble Aβ_1–42_ (h) in the hippocampi and cortices from APP/PS1 mice. (i–n) MN‐08 decreases Aβ accumulation and accelerates Aβ degradation of 3×Tg‐AD mice in the treatment experiment. (i and j) The total concentration of soluble Aβ_1–40_ (i) and insoluble Aβ_1–40_ (j) in the hippocampal and cortical regions from 3×Tg‐AD mice was variably reduced by treatment with MN‐08 (6 mg/kg), memantine (5 mg/kg), or donepezil (1.3 mg/kg). (k and l) The total concentration of soluble Aβ_1–42_ (k) and insoluble Aβ_1–42_ (l) in the hippocampi and cortices of 3×Tg‐AD mice. (m and n) Western blots and quantitative analyses of APP, sAPPα, NEP, and ADAM 10 in the hippocampi of similarly treated 3×Tg‐AD mice. Data are means ± SEM (*n* = 5–7). *^#^p* < 0.05 and *^##^p* < 0.01 versus control group; *p < 0.05, **p < 0.01 versus vehicle group, one‐way ANOVA with Tukey's multiple comparisons test

Amyloid β (Aβ) deposition is a typical pathological change seen in AD patients (Kennedy et al., 2016). We evaluated the deposition of Aβ_1‐42_ and Aβ_1‐16_ using immunohistochemical and immunofluorescence staining in the brains of APP/PS1 mice, respectively. Compared with non‐transgenic mice, APP/PS1 mice displayed considerable Aβ plaque deposition in both the hippocampal and cortical regions (Figure 3a–d). However, the APP/PS1 mice treated with MN‐08 and memantine in the prevention study had obviously attenuated amyloid plaque burdens (Figure 3a–d). To examine whether MN‐08 inhibits the production of Aβ, the total concentrations of Aβ_1–40_ and Aβ_1–42_ were quantitatively measured by ELISA test. Although there was no significant difference in levels of Aβ_1–40_, both soluble and insoluble Aβ_1–42_ in the hippocampal and cortical regions of APP/PS1 mice were significantly decreased by MN‐08 treatment (Figure 3e–h). Thus, these findings indicate that preventive administration of MN‐08 can reduce plaque deposition and total levels of Aβ in APP/PS1 mice.

### 2.3 MN‐08 alleviates Aβ accumulation and accelerates Aβ degradation in 3×Tg‐AD mice

Based on the above findings that the preventive administration of MN‐08 can attenuate Aβ burden in the brains of APP/PS1 mice, we next examined the treatment effects of MN‐08 on senile plaque deposition in 3×Tg‐AD mice. Similarly, in the ELISA tests, we observed a significant reduction in Aβ accumulation by MN‐08 treatment in the hippocampal and cortical areas of 3×Tg‐AD mice (Figure 3i–l). Mechanistically, MN‐08 treatment significantly decreased the expression of total APP. Moreover, MN‐08 treatment enhanced the expression of α‐secretase (ADAM10) and Aβ‐degrading enzyme neprilysin (NEP), which accelerated the decomposition of Aβ and APP, increasing the levels of sAPPα in 3×Tg‐AD mice (Figure 3m,n). Furthermore, MN‐08 consistently suppressed Tau hyperphosphorylation induced by Aβ in the hippocampi of APP/PS1 mice (Figure S2a,b). However, MN‐08 had no effect on the expression of insulin‐degrading enzyme (IDE), presenilin 1 (PS1), or β‐site APP cleaving enzyme 1 (BACE1; Figure S2c,d). These results demonstrate that MN‐08 can inhibit Aβ aggregation and accelerate Aβ degradation possibly through activation of ADAM10 and NEP.

### 2.4 MN‐08 restores dendritic spines in AD mice

**FIGURE 4:**
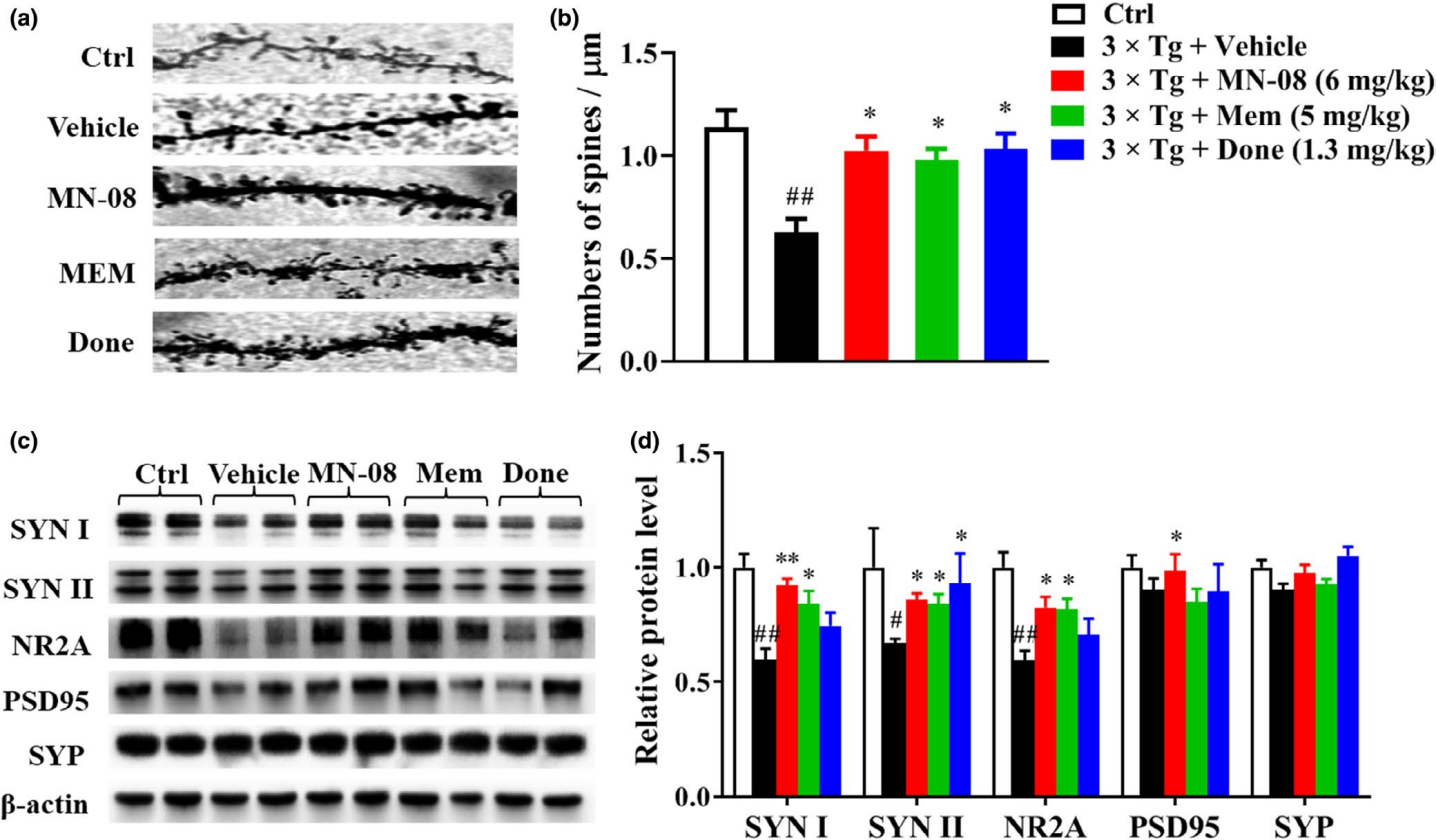
MN‐08 restores impaired dendritic spines in 3×Tg‐AD mice. (a and b) Representative photomicrographs (a) and quantitative analyses (b) of dendritic spines from hippocampal neurons (CA1) in 3×Tg‐AD mice treated with vehicle, MN‐08 (6 mg/kg), memantine (5 mg/kg), or donepezil (1.3 mg/kg). (c and d) Western blotting and quantitative analyses of synapse‐related proteins including synapsin I (SYN I), synapsin II (SYN II), NR2A, PSD95, and synaptophysin (SYP) in hippocampal from 3×Tg‐AD mice. Data are means ± SEM (*n* = 5–8). *^#^p* < 0.05 and *^##^p* < 0.01 versus control group; *p < 0.05, **p < 0.01 versus vehicle group, one‐way ANOVA with Tukey's multiple comparisons test

Hippocampal synaptic function and plasticity play a pivotal role in cognition and memory (Sala & Segal, 2014). We detected the density of dendritic spines by Golgi staining in the hippocampus CA1 area of 3×Tg‐AD mice. The number of dendritic spines was notably decreased in the vehicle‐treated 3×Tg‐AD mice compared with the normal control mice (Figure 4a,b). Importantly, this reduction of dendritic spines was markedly reversed by MN‐08, memantine, and donepezil (Figure 4a,b). To further confirm these findings, we next examined the synaptic markers, including synaptophysin (SYP), PSD95, synapsin I (SYN I), synapsin II (SYN II), and NR2A, using Western blotting. Vehicle‐treated 3×Tg‐AD mice showed a considerable reduction in these synapse‐associated proteins. Interestingly, the expression of synaptic markers was significantly increased by MN‐08 treatment (Figure 4c,d). Consistent with the data in 3×Tg‐AD mice, the APP/PS1 mice treated with MN‐08 in the prevention study also experienced a reversal of the depressed expression of synapse‐related proteins, including synaptophysin (SYP), PSD95, synapsin I (SYN I), synapsin II (SYN II), and drebrin (Figure S3a,b). These results suggest that MN‐08 can prevent synaptic loss and promote synaptic plasticity by regulation of synaptic proteins.

### 2.5 MN‐08 prevents glutamate‐induced excitotoxicity in CGNs

Excessive glutamate‐induced excitotoxicity might cause dysfunctions of cellular pathways, subsequently resulting in synaptic dysfunctions and neuronal loss. To evaluate the neuroprotective effects of MN‐08 against excitotoxicity, cerebellar granule neurons (CGNs) were treated with MN‐08 at the concentrations from 1 to 50 µM or memantine from 1 to 10 μM for 2 h, and then exposed to 100 μM glutamate for 24 h. MN‐08 could concentration‐dependently protect against glutamate‐induced excitotoxicity (Figure S4). Furthermore, as observed from phase‐contrast microscopy and Hoechst staining, after 24 h glutamate challenge, MN‐08 (30 µM) and memantine (10 µM) significantly reduced the numbers of apoptotic bodies and reversed the morphological changes, including the unhealthy shrieked cell bodies and broken neuritis network (Figure S5a,b). In addition, Western blot assay shown that pre‐treatment MN‐08 also significantly reversed the Bcl‐2 downregulation caused by glutamate (Figure S5c). Similarly, in APP/PS1 mice, MN‐08 prevented neuronal loss in the hippocampus by upregulating the expression ratio of Bcl‐2/Bax and downregulating the expression ratio of cleaved Caspase‐3 to Caspase‐3 (Figure S6).

### 2.6 MN‐08 reverses the dysregulations of ERK and PI3K/Akt/GSK3β pathway induced by glutamate

**FIGURE 5:**
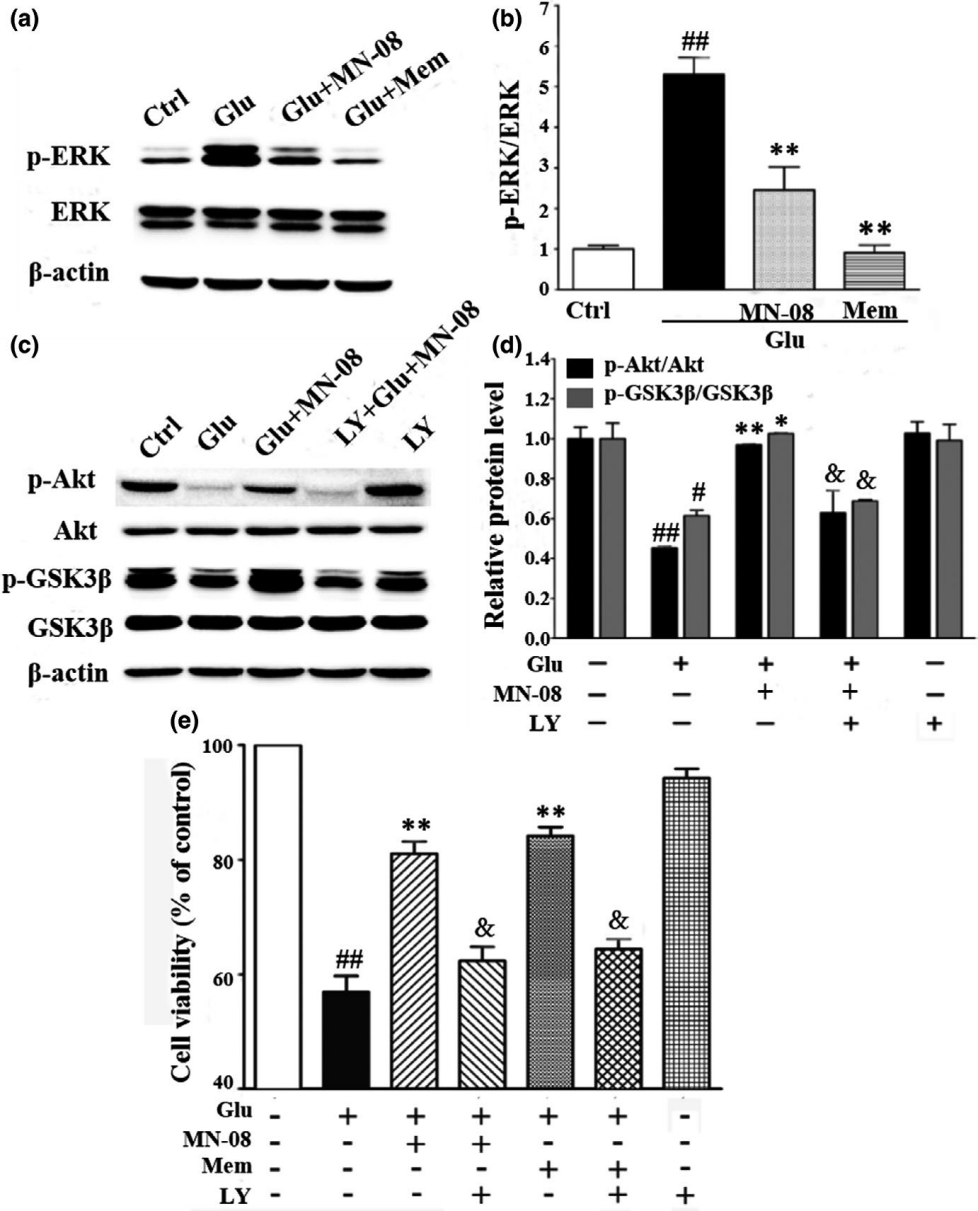
MN‐08 reverses the dysregulations of ERK and PI3K/Akt/GSK3β pathway induced by glutamate. CGNs were pre‐treated with MN‐08 (30 µM) and Mem (10 µM) for 2 h and then exposed to glutamate (100 µM) for 0.5 h, and the total proteins were extracted for Western blot analysis. (a and b) MN‐08 decreased glutamate‐induced the upregulation of p‐ERK. (c and d) Western blotting and quantitative analyses of p‐Akt/Akt and p‐GSK3β/GSK3β in CGNs. MN‐08 reversed the downregulation of p‐Akt and p‐GSK3β induced by glutamate. (e) Cell viability was examined using MTT assay. LY294002, a PI3K specific inhibitor, partially abolished the neuroprotection of MN‐08 through inhibiting PI3K/Akt/GSK3β pathway. The data represent the means ± SEM of three independent experiments. *^#^p* < 0.05 and *^##^p* < 0.01 versus control group; *p < 0.05 and **p < 0.01 versus glutamate alone group, ^&^ *p* < 0.05 versus glutamate plus MN‐08 group, one‐way ANOVA with Tukey's multiple comparisons test

Several studies have indicated that ERK and PI3K/Akt/GSK3β signal pathway might play a crucial role in glutamate‐induced excitotoxicity (Li et al., 2005; Llorens‐Martín et al., 2014). To further characterize the neuroprotection of MN‐08, the level of the key signaling molecules, such as ERK, Akt, and GSK3β, was examined by Western blotting assay. As shown in Figure 5a,b, thirty mins after glutamate incubation, the expression of p‐ERK was significantly increased compared with that of the control group. Pre‐treatment of MN‐08 (30 µM, 2 h) or memantine (10 µM, 2 h) significantly inhibited the upregulation of ERK1/2 phosphorylation induced by glutamate. Moreover, pre‐treatment with MN‐08 reversed expression of both p‐Akt and p‐GSK3β with the challenge of glutamate. Akt is an important downstream member of PI3K. To further confirm whether the PI3K/Akt pathway was associated with the neuroprotection of MN‐08 against glutamate‐induced excitotoxicity, specific PI3K inhibitor LY294002 was applied and incubated with MN‐08. It was found that LY294002 significantly abolished the upregulation of both p‐Akt and p‐GSK3β by the treatment of MN‐08 (Figure 5c,d). Additionally, the MTT assay showed that the neuroprotective effects of MN‐08 against glutamate‐induced apoptosis were canceled by LY294002, which might further confirm that PI3K/Akt/GSK3β were involved in the neuroprotective effect of MN‐08 (Figure 5e). Taken together, MN‐08 might protect against glutamate‐induce cytotoxicity by reversing the dysregulations of ERK pathway and PI3K/Akt/GSK3β pathway.

### 2.7 MN‐08 inhibits NMDA‐mediated current in primary hippocampal neurons

MN‐08, a memantine nitrates, was retained the backbone of memantine. We speculated that MN‐08 might also be capable to block the NMDA receptors. Firstly, molecular docking simulation was performed to investigate the binding effect of MN‐08 to NMDA receptors.

**FIGURE 6:**
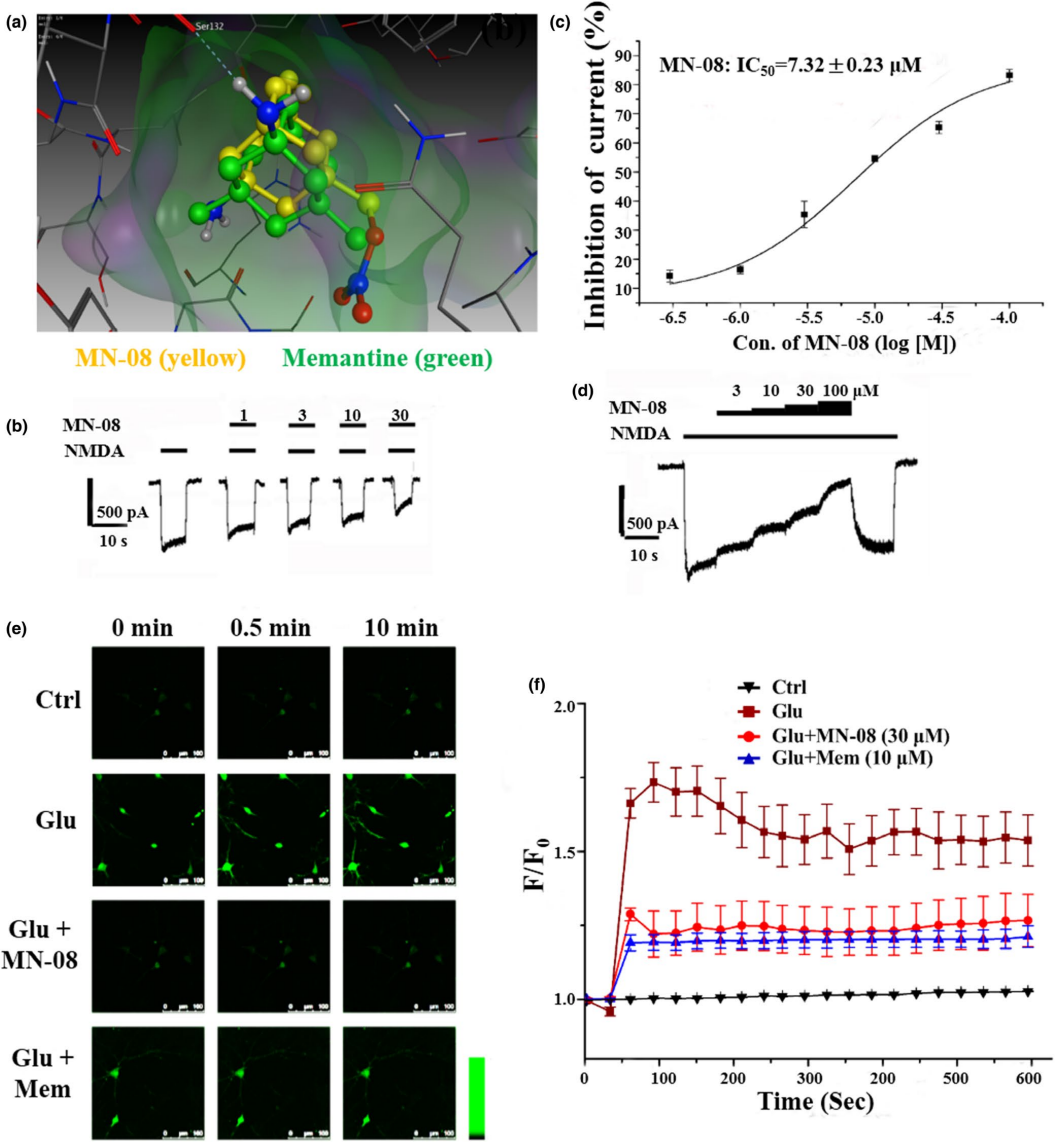
MN‐08 inhibits the NMDA‐mediated current and the glutamate‐induced Ca^2+^ increase in primary hippocampal neurons. (a) Possible acting site of MN‐08 and memantine (MEM) on NMDA receptor. A close‐up view of the low energy poses of MN‐08 (yellow) and memantine (green) in the NMDA receptor channel pore generated by molecular docking. (b) The representative current traces of the inhibition of NMDA‐mediate current by MN‐08 at different concentration from 1 to 30 μM. (c) The concentration‐response curve showing the inhibitory effect by MN‐08 on current mediated by NMDA (30 μM). (d) The representative current trace recorded by whole‐cell recording mode from the same patch‐clamped neuron. Current traces showing the control current induced by NMDA (30 μM) and the cumulative inhibitions incurred by four consecutive co‐applications of NMDA and MN‐08 (3–100 μM) at −50 mV. When the NM‐08 was washed out, the NMDA‐activated current was recovered. (e) The representative figures of live fluorescence intensity taken with a confocal microscope from hippocampal neurons exposed to glutamate (100 μM) with MN‐08 (30 μM) or Mem (10 μM) at different times. Scale bar, 100 μm. (f) The time course curves were constructed based on the fluorescence intensity determined by confocal microscope from primary hippocampal neurons exposed to the respective treatments. (F, the fluorescence value after exposure to glutamate within 10 min; F_0_, the fluorescence value just before exposure to glutamate). The data represent the means ± SEM of three independent experiments

The result showed that MN‐08 might antagonize NMDA receptors at the ion channel, with the free energy of binding of −6.3 kcal/mol. The free energy of binding of memantine with NMDA receptors is −5.4 kcal/mol. Additionally, as illustrated in Figure 6a, MN‐08 (yellow) interacted with NMDA receptors at the Ser132 residue, which is similar to memantine. Next, we examine the effects of MN‐08 on NMDA receptors by using electrophysiological technique. As shown in Figure 6b, it can be observed that MN‐08 (1–30 µM) inhibited NMDA‐activated current in rat hippocampal neurons by whole‐cell patch‐clamp recording. To determine the IC_50_, different concentrations of MN‐08 have been applied till the inhibitory effect reached steady state. The concentration responding curve constructed for MN‐08 is shown in Figure 6c; the calculated IC_50_ value of MN‐08 on the inhibition of NMDA‐mediated current is 7.32 ± 0.23 μM at holding potential of −50 mV. Moreover, NMDA (30 µM) was applied to induce NMDA‐mediated current, followed by MN‐08 at different concentration co‐applied with NMDA. It was found that the inhibitory effects were enhanced as the concentration of MN‐08 increased. Furthermore, when the application of MN‐08 was washed out, the NMDA‐activated current was recovered rapidly, suggesting that MN‐08 might be disassociated with NMDA receptors in a fast off‐rate (Figure 6d).

### 2.8 MN‐08 reduces intracellular Ca^2+^ in primary hippocampal neurons

NMDA receptors are highly permeable to Ca^2+^ in neurons. Overstimulation of NMDA receptors leads to excessive influx of Ca^2+^, which might be the important mediator of glutamate‐induced excitotoxicity by mediating the downstream pathways, including the activation of ERK and inhibition PI3K/Akt pathway. Thus, we further investigate whether MN‐08 could regulate the intracellular Ca^2+^ influx. Fluo4‐AM fluorescence probe was used for determining the Ca^2+^ levels in primary hippocampal neurons. The results demonstrated that the treatment of glutamate (100 µM) caused a rapid influx of Ca^2+^ in hippocampal neurons. With the treatment of MN‐08 (30 µM) or memantine (10 µM), the intracellular Ca^2+^ influx caused by glutamate was inhibited, evidenced by the reductions of fluorescence intensity (Figure 6e,f).

### 2.9 Pharmacokinetics and brain exposure, and safety evaluation of MN‐08 in vivo

The pharmacokinetic parameters for MN‐08 in rats are summarized in Table S1. After a single intragastric dose of 3, 12, or 24 mg/kg of MN‐08, the mean times to peak (*T*_max_) were 1.08, 1.83, and 4.00 h; the mean terminal half‐lives (*T*_1/2_) were 5.26, 6.81, and 6.82 h; and peak concentrations (*C*_max_) were 97.5, 399 and 674 ng/ml, respectively (Table S1). These results suggested that MN‐08 was rapidly absorbed from the gastrointestinal tract and distributed into tissues, and it possessed long terminal half‐life in healthy rats.

The brain exposure profiles of MN‐08 in SD rats were evaluated using a validated LC‐MS/MS method. When MN‐08 was administrated by gastric gavage to healthy rats at a single dose of 12 mg/kg, its concentration in the brain reached 5793 and 10,952 ng/g (approximately 19.92 and 37.67 μM, assuming the volume of 1 g brain tissue is 1 ml) at 0.5 and 1 h after drug administration, respectively (Table S2). Even after 24 h of administration, MN‐08 concentrations in the brain also reached 1661 ng/g (approximately 5.71 μM). Therefore, these results indicate that MN‐08 readily penetrates the blood‐brain barrier and achieves an effective therapeutic concentration.

In our toxicology study with beagle dogs, the no observed adverse effect level (NOAEL) of MN‐08 was 24 mg/kg by gastric gavage once daily for 1 month, while the NOAEL of memantine was 9 mg/kg. MN‐08 is thus likely safer and less toxic than memantine.

## 3 DISCUSSION

The safe and effective therapeutic strategies are urgently required for treatment of AD and yet almost all drug candidates have limited effects to improve cognitive functions in clinical trials (Selkoe & Hardy, 2016). In this study, MN‐08, a novel memantine nitrate, was able to, in accepted rodent models of AD, significantly reduced Aβ deposition, prevented neuronal and dendritic spine loss, all of which probably contributed to the significant observed attenuation of cognitive memory impairment seen in treated animals. In vitro, MN‐08 antagonized over‐activated NMDA receptors, inhibited calcium influx, and reversed the dysregulations of ERK and PI3K/Akt/GSK3β pathway, subsequently preventing glutamate‐induced neuronal loss. Furthermore, MN‐08 had favorable PK and safety profiles, in addition to penetrating the blood‐brain barrier to achieve effective therapeutic concentrations in the brain tissue. Taken in concert, the results of these animal studies suggest that MN‐08 is a promising drug candidate for AD treatment.

Overproduction and accumulation of Aβ in the brain are hypothesized to trigger the pathological cascades of AD (Hardy & Selkoe, 2002). APP/PS1 and 3×Tg‐AD mice begin to display amyloid plaques in the brain at 3 and 6 months of age, respectively (Zhong et al., 2014). We observed a considerable reduction in amyloid plaque deposition by preventatively treating APP/PS1 mice from ages 2 to 8 months with MN‐08. Next, we designed a therapeutic dosing protocol, the results of which showed that MN‐08 treatment effectively inhibited Aβ‐induced neurotoxicity in the brains of 8‐month‐old 3×Tg‐AD mice. Mechanistically, MN‐08 increased the activity of α‐secretase (ADAM10) and the expression of the Aβ‐degrading enzyme neprilysin (NEP), thus suppressing the amyloidogenic processing of APP and accelerating the degradation of already formed Aβ. Our previous evidence supported the notion that memantine selectively inhibited extrasynaptic over physiological synaptic NMDA receptor activity, protected synapses from Aβ‐induced damage both in vitro and in vivo (Talantova et al., 2013). In this study, novel memantine nitrates MN‐08 was found to restore dendritic spines and prevent synaptic loss in APP/PS1 and 3×Tg‐AD mice. Therefore, intake of MN‐08 before or after the onset of cerebral amyloid plaque deposition in AD model mice was able to reduce the Aβ burden, thereby attenuating synaptic damage and repairing cognitive impairment.

A reduction of CBF is found in patients with AD. Vascular dysfunction induced by Aβ deposition in the vessel walls of brain arterioles may be responsible for the decline of CBF in AD patients (Cruz Hernandez et al., 2019). When CBF decreases, there is a corresponding reduction in oxygen and glucose supply to the brain, which results in neuronal damage that can lead to permanent cognitive impairment (Justin et al., 2013; Marshall, 2012). Decreased CBF might be one of the main factors inducing the pathological progress of AD. NO was a potent vasodilator and regulator of CBF, and the levels of total NO were declined in patients with AD (Corzo et al., 2007). Memantine nitrates were designed by introducing the nitrate group into memantine moiety. These compounds would simultaneously inhibit NMDA receptors and release NO in the brain. Our previous study has confirmed that MN‐08 was able to dilate cerebral blood vessels and improve the CBF by binding NMDA receptors to release NO, ameliorating brain injury and cerebral vasospasm in SAH models (Luo et al., 2019).

Neuroprotection has long been the main focus of AD or brain aging studies due to the importance of neuron loss to cognitive deficits. Previous studies have reported that the glutamate‐induced excitotoxicity might cause neuronal death by the activation of ERK pathway and the suppression of PI3K/Akt pathways (Chen et al., 2018). In fact, the dysregulations of ERK and GSK3β have been found in a variety of in vitro and in vivo models associated with neurodegenerative diseases. However, MN‐08 could protect against glutamate‐induce cytotoxicity by reversing the dysregulations of ERK and PI3K/Akt GSK3β signal pathway. Interestingly, LY294002, a specific PI3K inhibitor might block the effects of MN‐08 on PI3K/Akt/ GSK3β signal pathway. The excessive influx of Ca^2+^ might be induced by overstimulation of NMDA receptors, which is crucial to initiate the apoptotic pathways, including ERK and PI3K/Akt pathways, and subsequently causes synaptic dysfunctions and neuronal loss (Bading, 2017; Hu et al., 2013). Our results have shown that MN‐08 could be capable to inhibit the Ca^2+^ influx by blocking the NMDA receptors. MN‐08 prevented neuronal loss and protected dendritic spine integrity and, most importantly, restored the cognitive impairment attributes of validated experimental models of AD. It is likely that the neuroprotective effect by MN‐08 is attributable to its multiple functions. Firstly, MN‐08 suppressed Aβ‐induced neurotoxicity; secondly, MN‐08 restored CBF by releasing exogenous NO and, finally, MN‐08 bound to and inhibited the over‐activation of neuronal NMDA receptors that can result in glutamate‐mediated excitotoxic neuronal damage.

Several NMDA receptors antagonists have been developed; however, few of them succeeded to pass the clinical trial and be the anti‐dementia drugs clinically due to the serious adverse effects (Alzheimer's, 2016). It has been revealed that many important physiological functions were mediated by NMDA receptors. Antagonists with high affinity could reduce the Ca^2+^ influx by antagonizing the NMDA receptors tightly, however, the normal physiological signal transductions might be also blocked. For example, MK‐801 and phencyclidine, which were excessively potent NMDA receptors antagonists with high affinity, cannot be used for treating dementia because they might affect the physiological functions of NMDA receptors (Coan et al., 1987). In our previous studies, memantine nitrates, which utilized the high‐affinity memantine binding site on NMDA receptor to target the NO group for interaction with the S‐nitrosylation/redox site external to the memantine binding site, were both well tolerated and effective against cerebral infarction in rodent models (Takahashi et al., 2015; Talantova et al., 2013). We found that the IC_50_ of the novel memantine nitrate MN‐08 on the inhibition of NMDA‐mediated current was slightly high than memantine. When the application of MN‐08 was washed out, the NMDA‐activated current was recovery rapidly. Thus, MN‐08 might be an ideal NMDA receptor antagonist, which is pathologically activated, uncompetitive with moderate affinity and fast off‐rate. In a toxicology study using beagle dogs, the NOAEL of MN‐08 was 24 mg/kg, while the NOAEL of memantine was 9 mg/kg. MN‐08 is thus less toxic than memantine. Moreover, our previous studies also found that MN‐08, despite containing a nitrate group, avoided causing side effects related to systemic hypotension while increasing cerebral blood flow (Luo et al., 2019). This unique trait may be attributable its PK characteristics: MN‐08 was rapidly absorbed and distributed into various tissues and organs, and the concentration of MN‐08 in brain tissue was much higher than that in the plasma.

In summary, we demonstrate that the novel memantine nitrate MN‐08 exerts a preventive and therapeutic effect in experimental animal models of AD. The features of the novel memantine nitrate MN‐08 that may confer benefit in AD are summarized as below: 1. Targeted delivery of the NO to the brain NMDA receptors by the memantine scaffold; 2. Dual site inhibition of NMDA receptors via the open‐channel block and NO/redox modulation of the receptor; 3. Cerebral vasodilation to increase CBF by binding NMDA receptors to release NO; and 4. Favorable PK and safety profiles. These promising findings justify clinical studies of MN‐08 in the treatment of neurodegenerative diseases, particularly Alzheimer's disease.

## 4 EXPERIMENTAL PROCEDURES

### 4.1 Study design

This study seeks to explore the efficacy and mechanism of action of MN‐08, a novel memantine nitrate, in established animal models of Alzheimer's disease. MN‐08’s effectiveness as a preventative and therapeutic agent was tested in 2‐ to 8‐month‐old APP/PS1 mice and 9‐ to 12‐month‐old 3×Tg‐AD mice, respectively. The neuroprotective mechanism of MN‐08 was tested in the glutamate cell model. The pharmacokinetics and safety of MN‐08 in vivo were determined in normal rats and beagle dogs. For the behavioral test, Western blotting analysis, pathology, ELISA test and in vitro cell tests, investigators were blinded to the experimental grouping and drug treatment. Sample sizes for each experiment are described in the figure captions.

### 4.2 Animal care

All mouse procedures were approved and supervised by the Shenzhen Center for Disease Control and Prevention Animal Welfare Committee (Shenzhen, China). Animals were housed in temperature‐ and humidity‐controlled rooms with a 12 h light/dark cycle, with free access to food and water throughout the experiment. All efforts were made to minimize the number of animals involved and ensure minimal suffering. The following animal models were used in this study.

### 4.3 APP/PS1 transgenic mice

APP/PS1 transgenic mice were purchased from Jackson Laboratory and reproduced in the Shenzhen Center for Disease Control and Prevention (Shenzhen, China). Two‐month‐old APP/PS1 mice were divided into four groups: (1) APP/PS1 + Vehicle group; (2) APP/PS1 + MN‐08 (6 mg/kg) group; (3) APP/PS1 + Memantine (Mem, 5 mg/kg) group; (4) APP/PS1 + Donepezil (Done, 1.3 mg/kg, once daily) group; (5) Two‐month‐old C57BL/6J mice as a control group. The APP/PS1 mice were administered drugs twice a day (Done, once daily) by gastric gavage from ages 2 to 8 months for 6 months successively. Control and vehicle groups received equal volumes of saline.

### 4.4 Triple transgenic 3×Tg‐AD mice

Triple transgenic 3×Tg‐AD mice were acquired from Jackson Laboratory and fostered in the Shenzhen Center for Disease Control and Prevention (Shenzhen, China). Eight‐month‐old 3×Tg‐AD mice were categorized into six groups: (1) 3×Tg‐AD +Vehicle group; (2) 3×Tg‐AD +MN‐08 (3 mg/kg) group; (3) 3×Tg‐AD +MN‐08 (6 mg/kg) group; (4) 3×Tg‐AD +MN‐08 (12 mg/kg) group; (5) 3×Tg‐AD +Memantine (Mem, 5 mg/kg) group; (6) 3×Tg‐AD +Donepezil (Done, 1.3 mg/kg, once daily) group; (7) Eight‐month‐old B6129SF2/J mice as a control group. MN‐08 and memantine were given twice daily (Done, once daily) by gastric gavage for 4 months from 8 months of age.

### 4.5 Healthy beagle dogs and rats

Healthy beagle dogs (males: weighing 6.43–8.40 kg; females: weighing 5.39–6.77 kg) and rats (weighing 220–250 g) were purchased from Beijing Marshall Biotechnology Co., Ltd. and were used to analyze MN‐08’s safety and PK characteristics, respectively. The beagle dogs and rats were housed at the animal facility of 3D BioOptima Co., Ltd. (License. No.: SYXK (Suzhou) 2016‐0040). The experimental procedures were approved by the Ethics Committee for Animal Experiments of 3D BioOptima Co., Ltd.

### 4.6 Statistical analysis

All data were expressed as mean ± SEM and were analyzed using GraphPad Prism 7 software (GraphPad software Inc.) and SPSS 13.0 statistic software (SPSS Inc.). Statistical analyses were conducted by one‐way or two‐way analysis of variance (ANOVA) followed by Tukey's test. *p* < 0.05 were considered statistically significant.

## CONFLICT OF INTEREST

Y.W.S., Y.Q.W., G.X.Z., and Z.J.Z. are share owners of Guangzhou Magpie Pharmaceuticals, LTD., Corp., who holds the patent covering the compound MN‐08. The other authors declare that no competing interests exist.

## AUTHOR CONTRIBUTIONS

Y.Q.W., Z.J.Z., and X.F.Y. designed and supervised the project. Z.Y.S., M.P.M.H., and Y.W.S. performed APP/PS1 mice experiments, collected and analyzed the data. X.H.Z., Y.W.C., and L.Z. performed 3×Tg‐AD mice experiments, collected and analyzed the data. L.M.W., N.L., S.H.M., and Y.F.H. performed the vitro experiments. L.M.W., X.F.Y., and G.X.Z. performed statistical analysis and prepared figure. L.M.W., Y.Q.W., and Z.J.Z. wrote the manuscript. All authors have read and approved the final manuscript.

## Supporting information

Supplementary Material

## Data Availability

The authors declare that the authors provide all data included in this study upon request when there is a reasonable request.
