# Supplementary material for: Therapeutic efficacy of novel memantine nitrate MN‐08 in animal models of Alzheimer’s disease
Source: Aging Cell. 2021 May 6;20(6):e13371. doi: 10.1111/acel.13371 (PMC8208785; doi:10.1111/acel.13371)
Supplement: Supplementary file 1 — Supplementary Material [file ACEL-20-e13371-s001.docx]

**Supporting information**

**Therapeutic efficacy of novel memantine nitrate MN-08 in animal models of Alzheimer’s disease**

**Running title: Therapeutic effect of MN-08 in AD**

Liangmiao Wu^1,†^, Xinhua Zhou^1,†^, Yiwan Cao^2^, Shing Hung MAK^3^, Ling Zha^2^, Ning Li^2^, Zhiyang Su^2^, Yifan Han^3^, Yuqiang Wang^2^, Maggie Pui Man Hoi^4^, Yewei Sun^2^, Gaoxiao Zhang^2,^*, Zaijun Zhang^2,^*, Xifei Yang^5,^*

^1^Department of Neurology and Stroke Center, The First Affiliated Hospital of Jinan University and Institute of New Drug Research, International Cooperative Laboratory of Traditional Chinese Medicine Modernization and Innovative Drug Development of Chinese Ministry of Education, Jinan University College of Pharmacy, Guangzhou 510632, China.

^2^Institute of New Drug Research, International Cooperative Laboratory of Traditional Chinese Medicine Modernization and Innovative Drug Development of Chinese Ministry of Education, Jinan University College of Pharmacy, Guangzhou 510632, China.

^3^Department of Applied Biology and Chemical Technology, Institute of Modern Chinese Medicine, The Hong Kong Polytechnic University, Hung Hom, Hong Kong, China.

^4^State Key Laboratory of Quality Research in Chinese Medicine and Institute of Chinese Medical Sciences, University of Macau, Macau 99078, China.

^5^Key Laboratory of Modern Toxicology of Shenzhen, Shenzhen Center for Disease Control and Prevention, Shenzhen 518000, China.

†These authors contributed equally to this work.

*Corresponding author. Email: [zaijunzhang@163.com](mailto:zaijunzhang@163.com) (Z.J.Z); zhanggaoxiao2005@163.com (G.X.Z); xifeiyang@gmail.com (X.F.Y).

This **Supporting information** includes:

**Experimental procedures**

**Supporting information Figure S1** MN-08 ameliorates cognitive deficits in AD mice evaluated by Morris Water Maze (MWM) test.

**Supporting information Figure S2** MN-08 alleviates Tau-Phosphorylation in APP/PS1 mice.

**Supporting information Figure S3** MN-08 activates synapse-associated proteins in APP/PS1 mice.

**Supporting information Figure S4** MN-08 protects against glutamate-induced neuronal death.

**Supporting information Figure S5** MN-08 attenuates the apoptotic hallmarks induced by glutamate in CGNs.

**Supporting information Figure S6** MN-08 reduces neuronal apoptosis in APP/PS1 mice.

**Supporting information Table S1** The pharmacokinetic parameters of MN-08 in rats.

**Supporting information Table S2** Mean concentrations of MN-08 (12 mg/kg) in brain (ng/g) and plasma (ng/mL) of rats.

**EXPERIMENTAL PROCEDURES**

**Step-down avoidance (SDA) test**

The step-down avoidance (SDA) test was performed as previously described ([Zhou et al., 2019](#_ENREF_14)). During the training phase, mice were singly placed on a metal floor with electrical stimulation (35 V) for 5 min. After 24 hours, the mouse was positioned on a wooden platform, while the metal floor at the bottom delivered an electrical shock (35 V) for another 5 min. The time for the animal to step down from the wooden platform were recorded as the step-down latency to evaluate retention memory.

**Morris water maze (MWM) test**

Hippocampus-dependent spatial memory was determined by the Morris water maze (MWM) test according to the protocol described in a previous study ([Jiao et al., 2015](#_ENREF_8)). The MWM test includes a platform training phase and a probe trial phase. An escape platform was placed in a fixed spatial location. During the training phase, each animal was placed at one of four different starting positions and tested four times a day with an interval of approximately 30 min. The animal in the water was immediately removed when it swam to the escape platform within 60 s. If the animal failed to find the escape platform by 60 s, it was gently guided to it. After the training phase, the probe trial was performed on the sixth day when the platform was removed. The animal was placed in the same starting position in the pool and allowed to move freely for 2 min. The animal swimming track was recorded and analyzed with a digital camera.

**Novel object recognition (NOR) test**

The novel object recognition test was performed to assess non-spatial working memory according to previously published protocols ([Choi et al., 2011](#_ENREF_3)). Firstly, each animal was sequentially placed into a square box (50 × 50 × 50 cm, length × width × height) without any objects for 10 min per day for 2 continuous days to habituate to the environment. Then, two identical objects were placed parallel near one wall of the square box. Animals were placed singly in the box and allowed to explore the objects for 10 min. After a one-hour interval, one of the old objects was replaced by a novel object and animals were allowed to explore for 5 min. The time each animal took to examine the familiar object and novel object was recorded by digital camera. The discrimination index was calculated according to the following expression: (novel object (s) - familiar object (s))/ (novel object (s) + familiar object (s)). The objects and the box were cleaned with ethanol (50 %) after each individual trial to eliminate olfactory cues.

**ELISA test of soluble and insoluble Aβ**

To evaluate the concentration of soluble and insoluble Aβ in AD mice, the hippocampus and cortex were homogenized in the lysate, which was then centrifuged at 12000 g for 30 min at 4°C. Supernatant samples were collected as soluble Aβ. Sediment of the cortex and hippocampus was resuspended with formic acid, incubated on ice for 60 min and then centrifuged at 12,000 g for 30 min at 4°C. The supernatant and Tris/NaH_2_PO_4_ buffer were mixed as insoluble Aβ. The concentration of Aβ was analyzed by ELISA kit of human amyloid β (R&D Systems, Minneapolis, MN, USA) following the manufacturer’s instructions.

**Golgi Staining**

Golgi staining was used to determine hippocampal dendritic spine density by a Rapid Golgi Stain Kit (FD Neurotechnologies, Columbia, MD, USA) according to the manufacturer’s instructions. The brain was removed after the behavioral tests and incubated with impregnation solution in the dark at room temperature for 1 week. Impregnation solution was replaced with solution A and stored in the dark for 3 days. Brain sections (100 μm thickness) were placed on gelatin-coated microscope slides and then immersed in ddH_2_O. Solution B, Solution C, 80 % ethanol and xylene were used for 10 min, sequentially. The dendritic spines of hippocampal neurons were analyzed and counted in a blinded manner from six sections per animal by light microscopy (Olympus, Tokyo, Japan).

**Immunohistochemistry and immunofluorescence staining**

Immunohistochemistry and immunofluorescence staining were carried out as previously described ([Zhang et al., 2017](#_ENREF_13)). Coronal brain sections were incubated with 0.01 mmol/L sodium citrate (pH 6.0) and heated for 15 min. After cooling to room temperature, the sections were immersed in 3 % H_2_O_2_ for 10 min followed by blocking solution (10 % fetal bovine serum, 1 % bovine serum albumin and 0.1 % Triton X-100) for 1 h. Sections were then incubated with the following appropriate primary antibodies overnight at 4°C: anti-Aβ_1-42_ antibody (#ab10148, 1:1000, Abcam), anti-Aβ_1-16_ antibody (#803014, 1:1000, Abcam) and anti-NeuN antibody (#ab177487, 1:400, Abcam). After rinsing 3 times, the sections of immunohistochemistry staining were incubated with biotin-conjugated universal secondary antibody (Gene Tech, Shanghai, China). For immunofluorescence staining, the sections were incubated with appropriate secondary antibodies for 2 h at room temperature. Image J software was used to analyze the fluorescence intensity and cell counting from six slices at similar coronal positions of each animal; this was performed by a viewer blinded to the experimental group.

**Western blotting analysis**

Western blotting analysis was performed as previously described with minor modifications ([Huang, Li, & Nan, 2017](#_ENREF_6)). Proteins were extracted from hippocampal tissue and their concentrations determined by the BCA protein assay kit (Fdbio science, Hangzhou, China). The protein (20 μg) was separated on a 12% SDS-PAGE gel and then transferred to PVDF membranes. The membranes were blocked using 5 % nonfat dry milk for 2 h. The protein blots were incubated with the corresponding primary antibodies against p-Akt (#4060, 1:1000, Cell Signaling Technology), Akt (#4685, 1:1000, Cell Signaling Technology), BACE1 (#5606, 1:1000, Cell Signaling Technology), PS1 (#5643, 1:1000, Cell Signaling Technology), p-GSK3β (#5558, 1:1000, Cell Signaling Technology), GSK3β (#12456, 1:1000, Cell Signaling Technology), Bcl-2 (#3498, 1:1000, Cell Signaling Technology), Bax (#5023, 1:1000, Cell Signaling Technology), Cleaved caspase-3 (#9661, 1:1000, Cell Signaling Technology), Caspase-3 (#9662, 1:1000, Cell Signaling Technology), ADAM10 (#14194, 1:1000, Cell Signaling Technology), APP (#[ab241592](https://www.abcam.cn/app-1-antibody-ab241592.html), 1:1000, Abcam), sAPPα (#ab126723, 1:1000, Abcam), NEP (#ab216341, 1:1000, Abcam), Synapsin I (#ab64581, 1:1000, Abcam), Synapsin II (#ab76494, 1:1000, Abcam), NR2A (#ab124913, 1:2000, Abcam), PSD95 (#ab18258, 1:1000, Abcam), Synaptophysin (#ab32127, 1:1000, Abcam), IDE (#ab133561, 1:1000, Abcam), Drebin (#ab178408, 1:1000, Abcam) and Tau-5 (#ab80579, 1:2000, Abcam) overnight at 4°C. After washing 3 times, the membranes were incubated with the respective HRP-conjugated secondary antibody. ECL Western blotting Detection Reagents (Fdbio science, Hangzhou, China) were used to perform Chemiluminescence detection. Quantitative analysis of protein bands was conducted by the Carestream system (Carestream Health, Inc, USA).

**Primary rat cerebellar granule neuron cultures**

CGNs were prepared from the 8-day-old Sprague–Dawley rats as described in our previous publication ([Li et al., 2005](#_ENREF_9)). Briefly, animals were anesthetized and sacrificed, and their cerebella were collected. Then, the freshly collected cerebella were rinsed in cold Kreb’s buffer-BSA and digested with 0.025 % trypsin incubated in a 37°C for 15 min. The reaction was stopped, and the single cell suspension was obtained through pipetting up and down the sedimentary tissue. CGNs were seeded in basal modified Eagle’s medium (ThermoFisher Scientific, Waltham, MA, USA) supplemented with 10 % fetal bovine serum, 25 mM KCl, 2 mM glutamine and 100 units/mL penicillin/streptomycin at a density of 1.5×10^6^ cells/mL. Cytosine arabinoside (10 μM) was added to inhibit the growth of non-neuronal cells at 24 h after seeding. All experiments were carried out after 8 days *in vitro*. All experimental procedures were performed according to the institutional animal experimental ethical guidelines at the Hong Kong Polytechnic University.

**Primary rat hippocampal neuron cultures**

Rat hippocampal neurons were obtained from 18-day-old Sprague-Dawley rat embryos as previously described with modifications ([Subramaniam et al., 2005](#_ENREF_11)). The hippocampi were obtained and dissected on ice. Then, the hippocampi were digested with 0.25 % trypsin at 37°C for 15 min. The single neurons were mechanically dissociated by using a Pasteur pipette. The neurons were plated at a density of 2×10^5^ cells/mL on 35-mm culture dishes, which were pre-coated with poly-L-lysine (10 μg/mL), in neurobasal eagle’s medium containing 10 % fetal bovine serum, 0.5 mM glutamine, 100 units/mL of penicillin, 100 μg/mL of streptomycin. The neurons were incubated under a humidified atmosphere of 5 % CO_2_, 95 % air at 37°C. In 24 h after the seeding, the culture medium was half-charged with neurobasal Eagle’s medium containing 2 % B27, 0.5 mM glutamine, 100 units/mL of penicillin, 100 μg/mL of streptomycin. The culture was half-changed with fresh medium twice weekly. Cells were used for confocal scanning 16 days after plating.

**Determination of cell viability**

Neurotoxicity was assessed using the tetrazolium salt 3-(4,5-dimethylthiazol-2-yl)-2,5- diphenyltetrazolium bromide dye (MTT) assay as previous publication ([Luo et al., 2010](#_ENREF_10)). Briefly, cells were cultured in 96-well plates. Then, different tested compounds at different concentrations were added and challenged by glutamate. After 24 h incubation, 10 μL of 5 mg/mL MTT was added to the medium. The cells were then incubated at 37 °C for another 4 h. After the removal of culture medium and then 100 μL of DMSO was added to dissolve the resulted formazan in each well. Cell viability was evaluated by observing colorimetric changes using a CLARIOstar plus Microplate Reader (BMG Labtech, Ortenberg, Germany) at a test wavelength of 570 nm with 655 nm as a reference wavelength. Data were expressed as a percentage of control cultures.

**Measurement of intracellular Ca^2+^ by confocal laser scanning microscopy**

A confocal laser scanning microscope was used to evaluate relative changes in intracellular calcium concentrations [Ca^2+^]_i_ by monitoring Fluo-4 fluorescence after intracellular cleavage of superfused Fluo-4 acetoxymethylester (1 μM, with excitation at 488 nm and emission at 510 nm) ([Bkaily, Al-Khoury, Simon, & Jacques, 2017](#_ENREF_1)). In brief, the neurons were stained with 1 μM Fluo-4 acetoxymethylester for 30 min in a 37°C incubator and then washed three times with a balanced salt solution containing 130 mM NaCl, 3 mM KCl, 1.25 mM Na_2_HPO_4_, 2 mM CaCl_2_, 26 mM NaHCO_3_, 10 mM glucose, pH 7.4. The fluorescence images were obtained by Leica TCS SPE Confocal Microscope and analyzed using the LAS AF software (Leica Microsystems Co., Wetzlar and Mannheim, Germany). The data were obtained by evaluating the fluorescence (F) from selected areas within a cell, following subtraction of background fluorescence, and division by the fluorescence intensity before drug application (F_0_), expressed as F/F_0_. Confocal images were taken and stored every 30 s. Drugs were added to the balanced salt solution 30 min prior to glutamate.

**Neuronal apoptosis determination by Hoechst 33342 staining**

Chromatin condensation, the apoptotic characteristic feature, was determined by Hoechst 33342 staining as described in previous publications with some modifications ([Guo et al., 2017](#_ENREF_4); [Jiajia et al., 2017](#_ENREF_7)). CGNs (1.5×10^6^ cells/mL) grown in a 6-well plate were rinsed with ice-cold PBS containing 5 % glucose. Then Hoechst 33342 (5 μg/mL) was added to the medium and incubated for 5 min. The nuclei were visualized by using a fluorescence Leica TCS SPE confocal (Leica, Wetzlar, Germany) at 200× magnification. In the observation, cells with bright blue fragmented nuclei, which were showing condensation of chromatin, were counted as apoptotic cells. Condensed nuclei were scored by counting at least 500 cells of three randomly chosen fields for each sample in three separated experiments.

**Whole-cell patch clamp recording**

Whole-cell patch clamp recordings were carried out at room temperature (22-24°C) on the stage of an inverted phase-contrast microscope using an Axopatch 700B patch amplifier (Axon Instruments, Burlingame, CA). Before each experiment, the culture medium was removed, the cells were rinsed completely and continuously superfused with a solution containing: 150 mM NaCl, 5 mM KCl, 0.25 mM CaCl_2_, 10 mM glucose, 0.001 mM glycine, 0.001 mM tetrodotoxin, 0.01 mM (-)-bicuculline methiodide and 10 mM HEPES (the pH was adjusted to 7.4 with NaOH, and the osmolarity was adjusted to ~340 mOsm with sucrose). The low concentration of Ca^2+^ was used to minimize the calcium-dependent desensitization of NMDA-activated current. Pipettes pulled from borosilicate glass (TW-150F, World Precision Instruments, Sarasota, FL, U.S.A.) had resistances of 2-4 MΩ when filled with pipette solution containing: 140 mM CsCl, 10 mM EGTA, 10 mM HEPES, and 5 mM MgATP with pH 7.3 (adjusted with CsOH) and 315 mOsm in osmolarity (adjusted with sucrose). A small patch of membrane underneath the tip of the pipette was aspirated to form a gigaseal and then a more negative pressure was applied to rupture it, thus establishing a whole-cell configuration. The adjustment of capacitance compensation and series resistance compensation was done before recording the membrane currents. The holding potential was set at –50 mV, except when indicated specially. Data were acquired on a computer using a DigiData interface and the pClamp9.0 software (Axon Instruments). Currents were filtered at 2 kHz and digitized at 5 kHz.

**Pharmacokinetic analysis of MN-08 in rats**

Healthy adult rats were cannulated under anesthesia one day before dosing, then randomly categorized into 3 groups (3 males and 3 females for each group): (1) MN-08 (3 mg/kg) group; (2) MN-08 (12 mg/kg) group; (3) MN-08 (24 mg/kg) group. Following single administration of MN-08 by gastric gavage, plasma samples (approximately 150 μL) were collected at different time points (Pre-dose and 0.25, 0.5, 1, 2, 4, 8, 12, 24 h after dose). The blood samples were placed in heparinized tubes and centrifuged at 3000 rpm for 10 min. The supernatant plasma was stored at -80°C until analysis. The concentration of MN-08 in blood samples was analyzed by the LC-MS/MS method as previously described ([Tian et al., 2016](#_ENREF_12)).

**Brain exposure of MN-08 in rats**

Bio-distribution studies were carried out in healthy adult rats (3 males and 3 females). All rats were administered MN-08 at a single dose of 12 mg/kg by gastric gavage. Brain and plasma were collected at 0.5, 1 and 24 h post dosing. Tissue samples were homogenized and then centrifuged at 12000 rpm for 10 min. The supernatants were injected into the LC-MS/MS system for analysis of MN-08 concentration according to the method previously described ([Tian et al., 2016](#_ENREF_12)).

**Toxicology study of MN-08 in beagle dogs**

Forty healthy adult beagle dogs were randomly divided into 4 groups of 5 males (weighing 6.43-8.40 kg) and 5 females (weighing 5.39-6.77 kg) each: (1) Placebo group; (2) MN-08 (4 mg/kg) group; (2) MN-08 (12 mg/kg) group; (3) MN-08 (24 mg/kg) group. MN-08 tablets were administered to beagle dogs by gastric gavage for 4 weeks. The no observed adverse effect level (NOAEL) was examined according to previously described ([Brownawell, Carmines, & Montesano, 2011](#_ENREF_2)).

**Supporting information FIGURES**


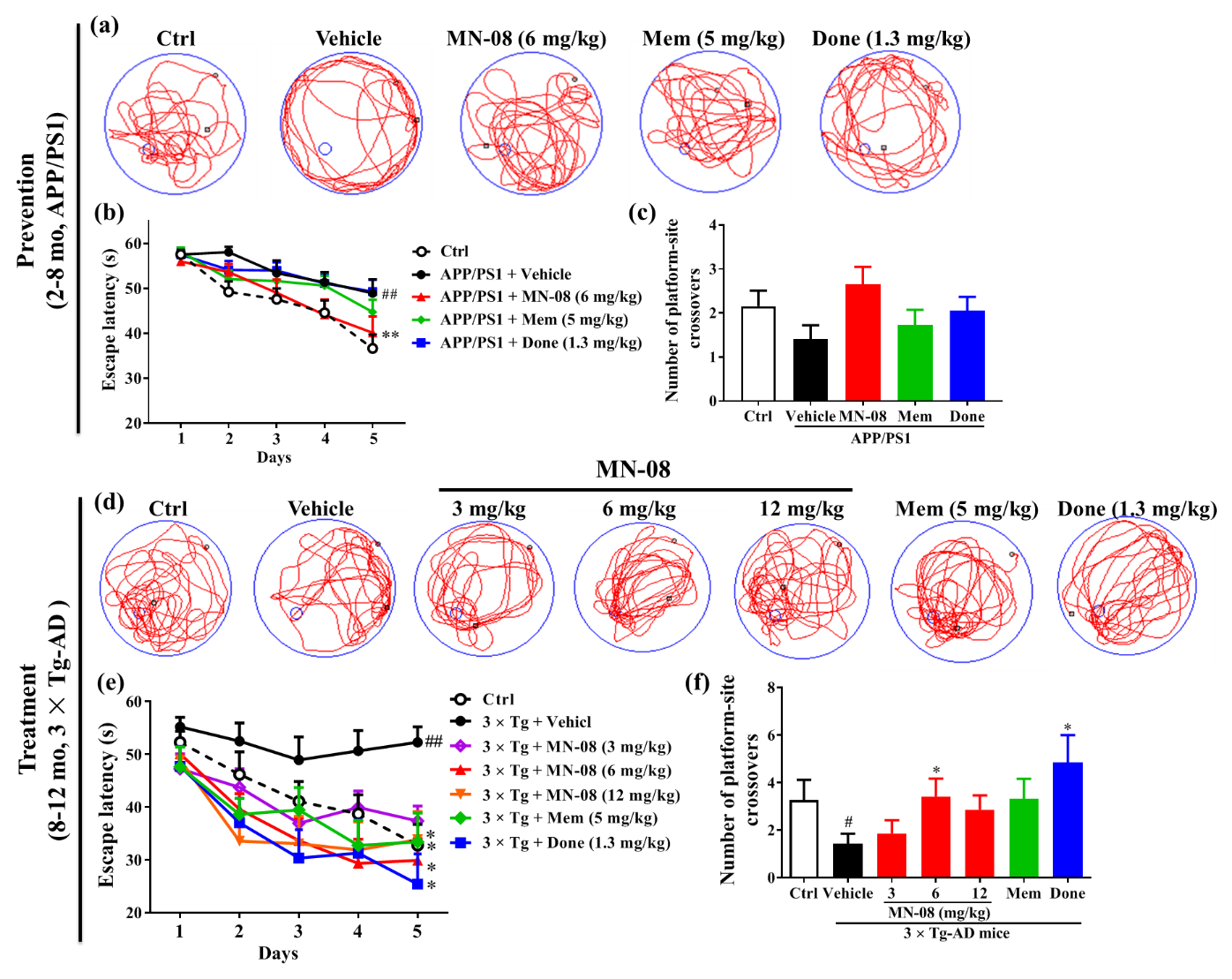


**Supporting information Figure S1 MN-08 ameliorates cognitive deficits in AD mice evaluated by Morris Water Maze (MWM) test. (a** to **c)** MWM test and quantitative analysis in MN-08’s preventative experiment of 2-month-old APP/PS1 mice (n=16 to 20). **(d** to **f)** MWM test and quantitative analysis in MN-08’s therapeutic experiment of 8-month-old 3×Tg-AD mice (n=11 to 14). **(a** and **d)** Representative swim traces during probe test (Day 6). **(b** and **e)** Escape latency during platform trials (Day 1 to Day 5). **(c** and **f)** Number of annulus crossing in the MWM probe phase (Day 6). Data are presented as mean ± SEM. Significance was determined by two-way **(b** and **e)** or one-way **(c** and **f)** ANOVA followed by Tukey’s multiple comparisons test. ***^#^****P* < 0.05 and ***^##^****P* < 0.01 vs. control group; **P* < 0.05, ***P* < 0.01 vs. vehicle group.


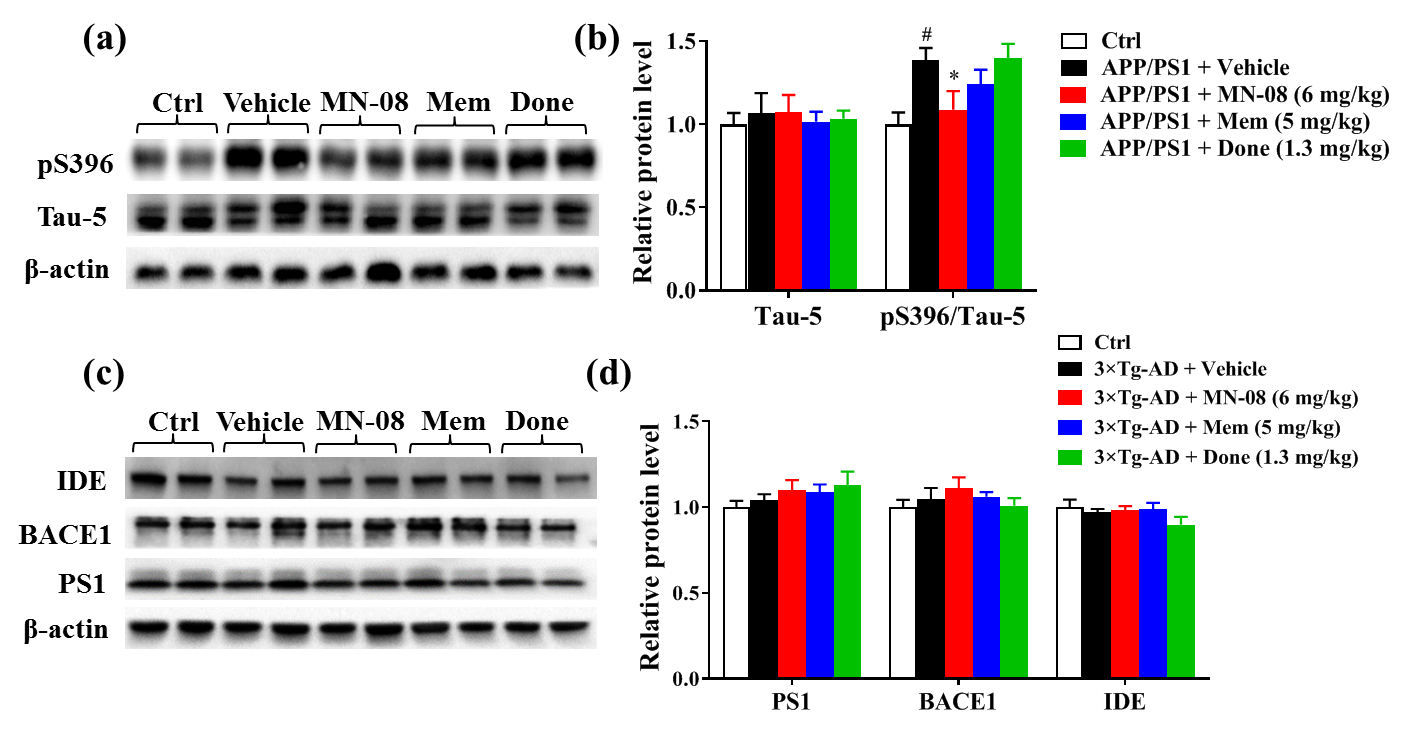


**Supporting information Figure S2** MN-08 alleviates Tau-phosphorylation in APP/PS1 mice. (**a** and **b**) Representative protein band (**a**) and quantitative analyses (**b**) of Tau-phosphorylation and Tau-5 in hippocampi from APP/PS1 mice. (**c** and **d**) Representative band (**c**) and quantitative analyses (**d**) of IDE, BACE1 and PS1 in hippocampi from 3×Tg-AD mice. Data are means ± SEM (n=4). ***^#^****P* < 0.05 vs. control group; **P* < 0.05 vs. vehicle group, one-way ANOVA with Tukey’s test.


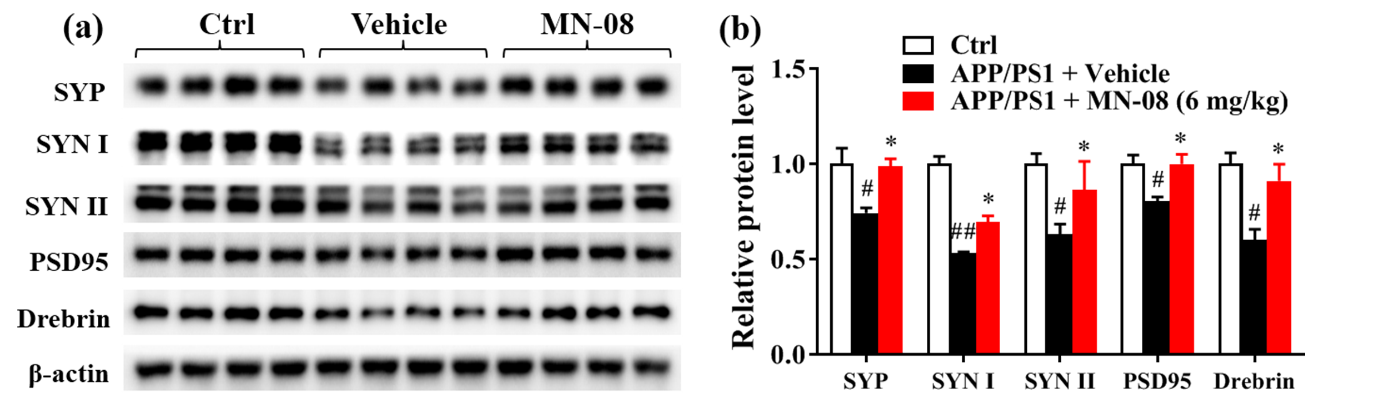


**Supporting information Figure S3** MN-08 activates synapse-associated proteins in APP/PS1 mice. (**a** and **b**) Representative protein band (**a**) and quantitative analyses (**b**) of synaptophysin (SYP), synapsin I (SYN I), synapsin II (SYN II), PSD95 and Drebrin in hippocampal homogenates. Data are means ± SEM (n=4). ***^#^****P* < 0.05 and ***^##^****P* < 0.01 vs. control group; **P* < 0.05 vs. vehicle group, one-way ANOVA with Tukey’s test.


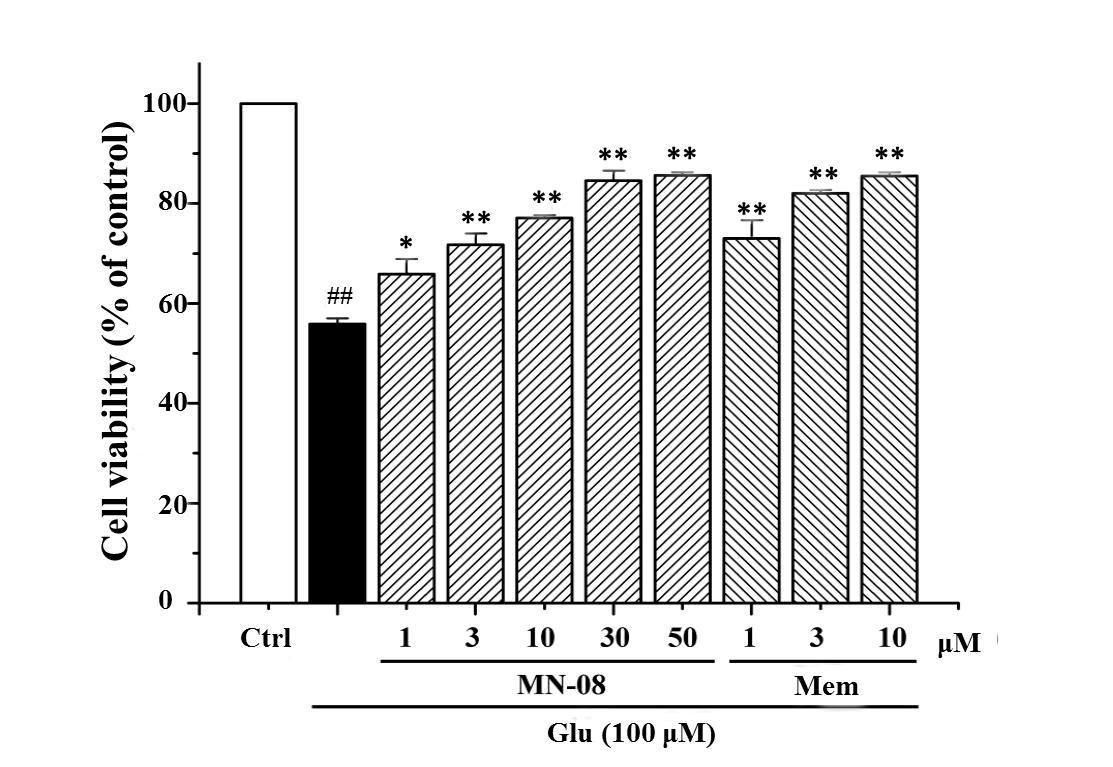


**Supporting information Figure S4** MN-08 protects against glutamate-induced neuronal death. CGNs were pre-treated with MN-08 (1-50 µM) or memantine (1-10 μM) for 2 h, and then incubated with 100 µM glutamate for additional 24 h, and finally subjected to MTT assay. Data were the mean ± SEM of three separate experiments. ***^##^****P* < 0.01 vs. control group; **P* < 0.05 and ***P* < 0.01 vs. glutamate alone group, one-way ANOVA with Tukey’s test.


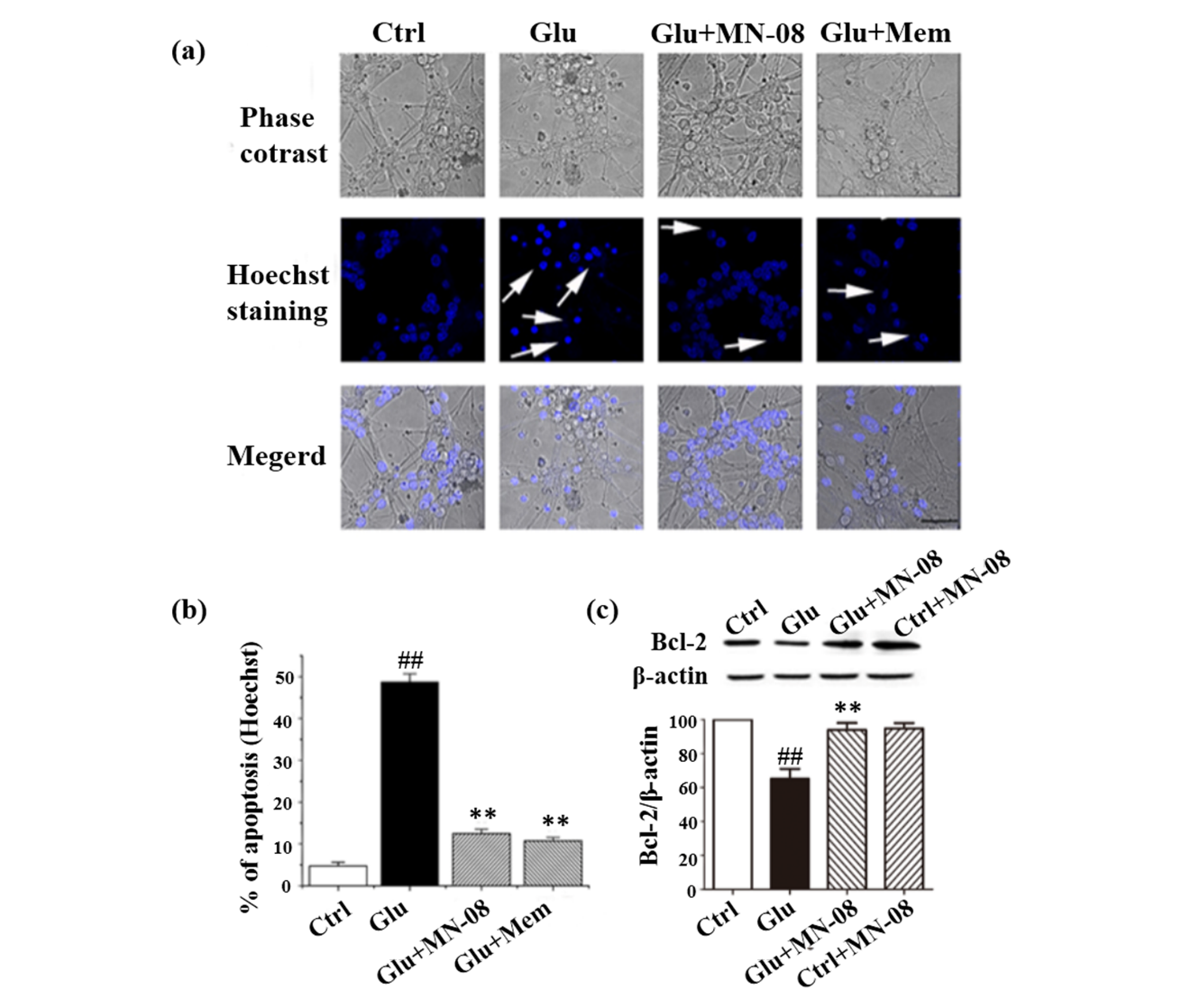


**Supporting information Figure S5** MN-08 attenuates the apoptotic hallmarks induced by glutamate in CGNs. CGNs were pre-treated with or without MN-08 (30 μM) or Mem (10 μM) for 2 h, and then exposed to glutamate (100 µM). After 24 h glutamate challenge, (**a**) Apoptotic cells that displayed bright blue nuclear condensations are indicated by arrows. The nuclear condensations were pointed by arrows. (**b**) Quantitative analyses of apoptotic bodies by Hoechst staining. (**c**) Western blot of the apoptosis related protein Bcl-2. Data were the mean ± SEM of three separate experiments. ***^##^****P* < 0.01 vs. control group; ***P* < 0.01 vs. glutamate alone group, one-way ANOVA with Tukey’s test.

**
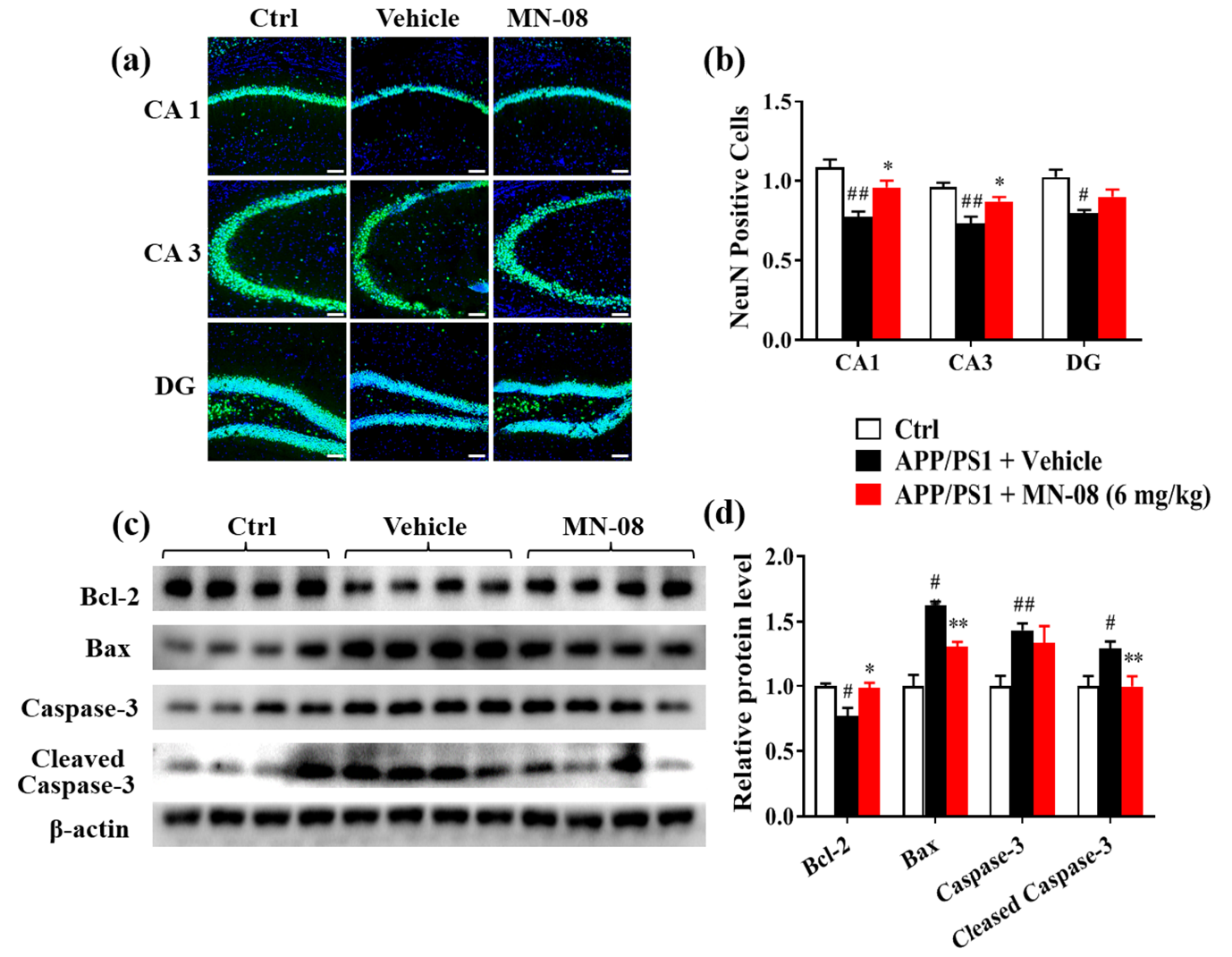
**

**Supporting information Figure S6** MN-08 reduces neuronal apoptosis in APP/PS1 mice. (**a** and **b**) Representative photomicrographs (**a**) and quantitative analyses (**b**) of NeuN staining in hippocampal area from APP/PS1 mice. (**c** and **d**) Representative band (**c**) and quantitative analyses (**d**) of neuronal apoptosis-related protein in hippocampi from APP/PS1 mice, including Bcl-2, Bax, Caspase-3 and Cleaved Caspase-3. Data are means ± SEM (n=4). ***^#^****P* < 0.05 and ***^##^****P* < 0.01 vs. control group; **P* < 0.05 and ***P* < 0.01 vs. vehicle group, one-way ANOVA with Tukey’s test. Scale bar, 50 μm.

**Supporting information Table S1** The pharmacokinetic parameters of MN-08 in rats.

| Dose  (mg/kg) | T_1/2_  (h) | T_max_  (h) | C_max_  (ng/mL) |
| --- | --- | --- | --- |
| 3 | 5.26±0.71 | 1.08±0.49 | 97.5±13.2 |
| 12 | 6.81±1.21 | 1.83±0.41 | 399±51.0 |
| 24 | 6.82±0.77 | 4.00±3.29 | 674±95.1 |

Data are presented as mean ± SD (n=6).

T_1/2_, mean terminal half-lives; T_max_, mean times to peak; C_max_, peak concentrations.

**Supporting information Table S2** Mean concentrations of MN-08 (12 mg/kg) in brain (ng/g) and plasma (ng/mL) of rats.

| Tissues | 0.5 h | 1 h | 24 h |
| --- | --- | --- | --- |
| Brain | 5793±1033 | 10952±3236 | 1661±183.4 |
| Plasma | 781±99 | 726±141 | 73.4±23.8 |

Data are presented as mean ± SD (n=6).

**REFERENCES**

Bkaily, G., Al-Khoury, J., Simon, Y., & Jacques, D. (2017). Intracellular free calcium measurement using confocal imaging. *Methods Mol Biol, 1527*, 177-187. https://doi.org/10.1007/978-1-4939-6625-7_14

Brownawell, A. M., Carmines, E. L., & Montesano, F. (2011). Safety assessment of AGPC as a food ingredient. *Food Chem Toxicol, 49*(6), 1303-1315. https://doi.org/10.1016/j.fct.2011.03.012

Choi, B. R., Lee, S. R., Han, J. S., Woo, S. K., Kim, K. M., Choi, D. H., et al. (2011). Synergistic memory impairment through the interaction of chronic cerebral hypoperfusion and amlyloid toxicity in a rat model. *Stroke, 42*(9), 2595-2604. https://doi.org/10.1161/strokeaha.111.620179

Guo, B., Hu, S., Zheng, C., Wang, H., Luo, F., Li, H., et al. (2017). Substantial protection against MPTP-associated Parkinson's neurotoxicity in vitro and in vivo by anti-cancer agent SU4312 via activation of MEF2D and inhibition of MAO-B. *Neuropharmacology, 126*, 12-24. https://doi.org/10.1016/j.neuropharm.2017.08.014

Harada, H., Wang, Y., Mishima, Y., Uehara, N., Makaya, T., & Kano, T. (2005). A novel method of detecting rCBF with laser-Doppler flowmetry without cranial window through the skull for a MCAO rat model. *Brain Res Brain Res Protoc, 14*(3), 165-170. https://doi.org/10.1016/j.brainresprot.2004.12.007

Huang, Y., Li, Z., & Nan, G. (2017). Effect of hippocampal LNBP on BDNF and TrkB expression and neurological function of vascular dementia rats. *Mol Med Rep, 16*(5), 7673-7678. https://doi.org/10.3892/mmr.2017.7539

Jiajia, L., Shinghung, M., Jiacheng, Z., Jialing, W., Dilin, X., Shengquan, H., et al. (2017). Assessment of Neuronal Viability Using Fluorescein Diacetate-Propidium Iodide Double Staining in Cerebellar Granule Neuron Culture. *J Vis Exp*(123). https://doi.org/10.3791/55442

Jiao, S. S., Yao, X. Q., Liu, Y. H., Wang, Q. H., Zeng, F., Lu, J. J., et al. (2015). Edaravone alleviates Alzheimer's disease-type pathologies and cognitive deficits. *Proc Natl Acad Sci U S A, 112*(16), 5225-5230. https://doi.org/10.1073/pnas.1422998112

Li, W., Pi, R., Chan, H. H., Fu, H., Lee, N. T., Tsang, H. W., et al. (2005). Novel dimeric acetylcholinesterase inhibitor bis7-tacrine, but not donepezil, prevents glutamate-induced neuronal apoptosis by blocking N-methyl-D-aspartate receptors. *J Biol Chem, 280*(18), 18179-18188. https://doi.org/10.1074/jbc.M411085200

Luo, J., Li, W., Zhao, Y., Fu, H., Ma, D. L., Tang, J., et al. (2010). Pathologically activated neuroprotection via uncompetitive blockade of N-methyl-D-aspartate receptors with fast off-rate by novel multifunctional dimer bis(propyl)-cognitin. *J Biol Chem, 285*(26), 19947-19958. https://doi.org/10.1074/jbc.M110.111286

Subramaniam, S., Shahani, N., Strelau, J., Laliberte, C., Brandt, R., Kaplan, D., et al. (2005). Insulin-like growth factor 1 inhibits extracellular signal-regulated kinase to promote neuronal survival via the phosphatidylinositol 3-kinase/protein kinase A/c-Raf pathway. *J Neurosci, 25*(11), 2838-2852. https://doi.org/10.1523/JNEUROSCI.5060-04.2005

Tian, X., Li, H. M., Wei, J. Y., Liu, B. J., Zhang, Y. H., Wang, G. J., et al. (2016). Preclinical Pharmacokinetics, Tissue Distribution, and Plasma Protein Binding of Sodium (+/-)-5-Bromo-2-(alpha-Hydroxypentyl) Benzoate (BZP), an Innovative Potent Anti-ischemic Stroke Agent. *Front Pharmacol, 7*, 255. https://doi.org/10.3389/fphar.2016.00255

Zhang, T., Gu, J., Wu, L., Li, N., Sun, Y., Yu, P., et al. (2017). Neuroprotective and axonal outgrowth-promoting effects of tetramethylpyrazine nitrone in chronic cerebral hypoperfusion rats and primary hippocampal neurons exposed to hypoxia. *Neuropharmacology, 118*, 137-147. https://doi.org/ 10.1016/j.neuropharm.2017.03.022

Zhou, X., Xiao, W., Su, Z., Cheng, J., Zheng, C., Zhang, Z., et al. (2019). Hippocampal Proteomic Alteration in Triple Transgenic Mouse Model of Alzheimer's Disease and Implication of PINK 1 Regulation in Donepezil Treatment. *J Proteome Res, 18*(4), 1542-1552. https://doi.org/10.1021/acs.jproteome.8b00818

Zuloaga, K. L., Zhang, W., Yeiser, L. A., Stewart, B., Kukino, A., Nie, X., et al. (2015). Neurobehavioral and imaging correlates of hippocampal atrophy in a mouse model of vascular cognitive impairment. *Transl Stroke Res, 6*(5), 390-398. https://doi.org/10.1007/s12975-015-0412-z
